# Heterogeneous PMO@MXene nanocomposite: a robust and efficient nanocatalyst for two-component synthesis of highly substituted 2-(aryl)-1*H*-benzo[*d*]imidazole derivatives

**DOI:** 10.1038/s41598-025-28219-8

**Published:** 2025-11-28

**Authors:** Safa Hanifi, Farhad Shirini, Bahram Ramezanzadeh, Hassan Tajik

**Affiliations:** 1 Department of Organic Chemistry, Faculty of Chemistry, University of Guilan, Rasht, 41335-19141 Iran; 2 Department of Surface Coating and Corrosion, Institute for Color Science and Technology, Tehran, Iran

**Keywords:** PMO, MXene, Ti_3_AlC_2_, Mesoporous organosilica, Benzimidazle derivatives, Nanocomposite, Chemistry, Materials science, Nanoscience and technology

## Abstract

In this work, a novel MXene-based nanocomposite, denoted as PMO@MXene, was synthesized for the first time and successfully applied as an efficient heterogeneous catalyst in the green synthesis of 2-(aryl)-1*H*-benzo[*d*]imidazole derivatives. This catalyst has been used for the synthesis of new derivatives, which were characterized using ^13^CNMR, ^1^HNMR, TLC, and mass spectrometry. The hybrid material was fabricated through the integration of periodic mesoporous organosilica (PMO) onto the layered structure of MXene, combining the properties of PMO with the excellent conductivity and catalytic potential of MXene. Comprehensive characterization using XRD, FTIR, FESEM, BET, and HRTEM analyses confirmed the successful formation and structural integrity of the heterogeneous PMO@MXene nanocomposite. The catalytic activity of the PMO@MXene nanocomposite was investigated under mild and environmentally benign conditions, leading to high yields in short reaction times with minimal catalyst loading. Notably, the catalyst exhibited remarkable reusability over multiple cycles with negligible loss of performance. This study introduces the PMO@MXene nanocomposite as a promising, sustainable, and recyclable platform for efficient organic transformations, particularly for the synthesis of biologically significant benzimidazole scaffolds.

**Supplementary Information:**

The online version contains supplementary material available at 10.1038/s41598-025-28219-8.

## Introduction

In recent years, using different catalysts in various organic reactions has become a major concern for environmental sustainability. In this area, the reliance on toxic, non-recyclable, and expensive catalysts contributes to the challenges associated with these materials^1,2^.

Mesoporous materials have garnered significant attention in recent decades and are widely utilized in various fields, primarily due to their distinctive porous architecture and large surface area^3^. Porous materials are one of the best supports that can be used in the production of heterogeneous catalysts^4–6^. A recent advancement in the synthesis of mesoporous materials has focused on the cross-linking of various functional groups, including amines, strong acidic ionic liquids, sulfonates, triphenylphosphine, pyridine, and other similar compounds, onto porous structures^7,8^.

Periodic Mesoporous Organosilicas (PMOs) are an innovative type of organic-inorganic nanocomposites designed for various applications, including catalysis, sensing, separations, and microelectronics. A key feature of PMOs is the incorporation of organic bridging groups of the form Si-R-Si(OR^**/**^)_3_ (R^**/**^: methyl or ethyl; R: an organic functional group)^9,10^ within the walls of their ordered nanoporous structure. This design allows for the precise tuning of both the chemical and physical properties of the materials^11^. PMO materials can be synthesized through the sol-gel process using organo-bridged alkoxysilanes along with structure-directing agents^12^.

Following the discovery of the M41S silicas^13^, the synthesis of periodic mesoporous materials with varying compositions has become a prominent research focus in this area. Initial studies concentrated on modifying the silica framework by incorporating different cations, including Al^3+^, B^3+^, Ti^4+^, V^5+^, and others, aiming to develop acid catalysts or catalysts for selective oxidation^14–16^.

As we have mentioned previously, in early research on PMOs, the materials were synthesized using a single bridged silane framework. Subsequent studies employed two or more organosilane precursors to create PMOs with enhanced or multifunctional properties. These bridged organosilica precursors incorporated heteroelements such as nitrogen, sulfur, phosphorus, and oxygen, along with metal complexes and chiral bridges. Typically, these PMOs were produced in either powder or film morphologies^17^. Based on their structures, PMOs are highly significant for coupling and redox catalytic processes^18^. Additionally, PMOs with a large surface area and specific functional groups are highly effective adsorbents for heavy metal ions, toxic organic compounds, and pollutant gases in the environment. On the other hand, the surface of mesoporous silica can be tailored with different organic functional groups to create organic–inorganic hybrid materials for catalytic applications^19^. The recent focus on preparing organometallic complexes in a heterogeneous format has gained popularity due to its advantages for green catalysis. This approach addresses concerns about cost efficiency, the recovery and reusability of expensive or toxic catalysts, and the prevention of product contamination^20^.

The discovery of monolayer graphene in 2004 has generated substantial interest in two-dimensional (2D) materials^21,22^. Since their discovery in 2011, MXenes, which are 2D carbides, nitrides, and carbonitrides of early transition metals, have become one of the most extensively researched families of 2D materials^23^.

These compounds are considered the next generation of two-dimensional materials suitable for a wide range of applications. These include photocatalysis^24–27^, electrocatalysts^28,29^, supercapacitors^30,31^, lithium-ion batteries^32,33^, biomedicine, and catalysis, thanks to their exceptional physicochemical properties^34^.

MXene has a structural formula expressed as M_n+1_X_n_T_x_, where M denotes early transition metals like Ti, V, Zr, and Nb, X represents carbon and/or nitrogen, and T refers to the surface functional groups such as –O, –OH, and –F. This formula is achieved by etching the ‘A’ layer element in MAXphase ceramics, which represents the most recent addition to the 2D material family due to its relatively recent discovery^35^. Due to the variation in atomic layer numbers within the unit cell, the value of n can vary from 1 to 3, leading to the characteristic structures of M_2_XT_x_, M_3_ × _2_T_x_, and M_4_ × _3_T_x_, respectively^34^.

To date, over 30 different MXenes have been synthesized experimentally, including Ti_3_C_2_T_x_^36^, Mo_2_CT_x_^37^, Ti_2_CT_x_^38^, Nb_4_C_3_T_x_^39^, Nb_2_CT_x_^40^, V_2_CT_x_^41^, Mo_2_TiC_2_T_x_^42^, and Mo_2_Ti_2_C_3_T_x_^43^. Among these, Ti_3_C_2_T_x_ has been the most extensively studied^44^.

MXenes exhibit fascinating mechanical, electronic, magnetic, and electrochemical properties. These compounds also possess distinctive characteristics that suggest significant potential for biomedical applications (for example, fabrication of bio-scaffolds)^45,46^. Notably, their excellent flexibility, coupled with a two-dimensional morphology and layered structure, allows MXenes to combine easily with other materials. This capability presents an opportunity to integrate the distinctive properties of various materials in a complementary way^47,48^.

**Scheme 1:**
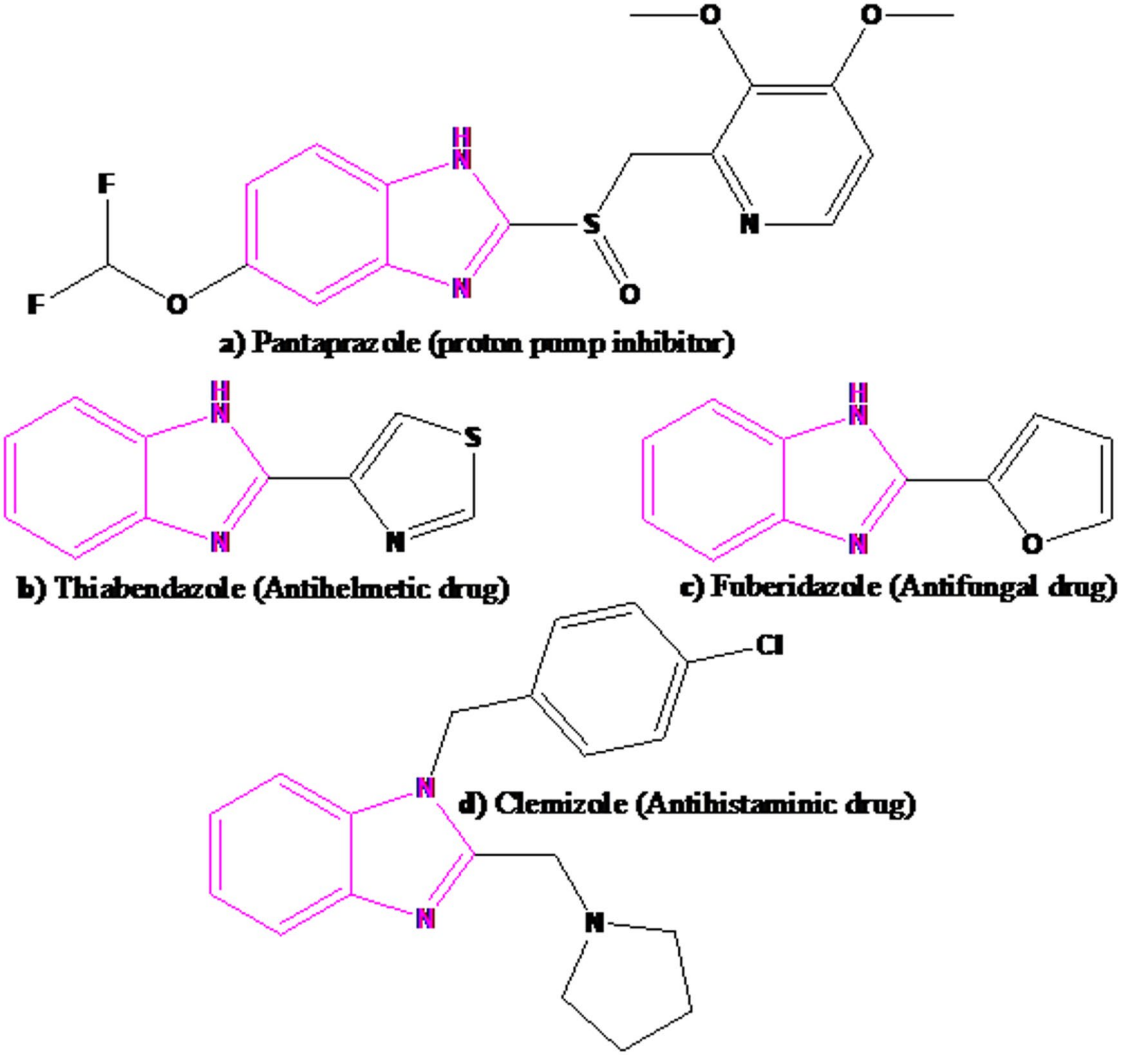
Commercial drugs containing benzimidazole ring.

Benzimidazoles and their derivatives represent an important category of heterocyclic compounds that exhibit notable biological activities^49,52^. These compounds are also a class of interesting pharmacophores with various useful pharmacological properties^53^ (Scheme 1). These include antitumor^54^, anti-inflammatory^55^, antimicrobial^56,57^, and anticancer^58^ effects. Also, they serve as key intermediates in the synthesis of various drugs; for instance, the structure of vitamin B12 includes a benzimidazole framework^59^. Because of their important characteristics, a variety of methods and catalysts have been reported for their synthesis^60–62^. These methods are also useful, but most of them include disadvantages such as long-time reaction, low yields, use of toxic reagents or solvents, use of expensive materials, non-recyclability of the catalyst, etc. So, the introduction of new catalysts which their use can solve all or some of these restrictions is still in demand.

Based on this idea that the combination of PMOs and MXenes in a composite can lead to synergistic effects, where the integrated properties of MXenes and PMOs enhance performance compared to their components so that the prepared composite can represent a significant advancement in materials science and chemistry, paving the way for next-generation applications in nanotechnology, electronics, and chemical reactions in this study, and for the first time, we wish to report the preparation and identification of a PMO@MXene nanocomposite and its use in the acceleration of the synthesis of 2-(aryl)-1-*H*-benzo[d]imidazole derivatives. The purpose of designing new derivatives is to investigate the higher efficiency of the catalyst in various aldehydes. The newly designed derivatives synthesized in this work have not been reported so far, and this article, by providing ^13^CNMR, ^1^HNMR data, and melting point demonstrates the successful synthesis of these compounds. The obtained results showed that this catalyst not only leads to considerable simplifications in the synthesis of the mentioned target molecules, but also solves some of the difficulties which are associated with the previously reported catalysts for some reactions.

## Experimental

All substrates, including ethanol, tetraethyl orthosilicate (TEOS), Pluronic P123, bis[3-(trimethoxysilyl) propyl] amine (BTPA), hydrochloric acid (HCl), lithium fluoride (LiF), MAXphase (Ti_3_CAl_2_), 3-aminopropyl triethoxysilane, and aldehydes, were purchased from Sigma, Aldrich, and Merck Chemical Companies. Additionally, pure distilled H_2_O and 96% EtOH were used as solvents. A UV lamp emitting light at a wavelength of 254 nm was utilized to conduct the thin-layer chromatography (TLC) experiment. The identification of the products was achieved through the use of a Shimadzu FTIR 8400 S spectrometer, employing potassium bromide disks. Furthermore, ^1^HNMR spectra were recorded using a Bruker Avance 500 in DMSO-d_*6*_ solvent at ambient temperature. The melting points were measured using a 9100 Electrothermal apparatus and were presented without any corrections. The reported yields are calculated based on the products obtained after the purification process.

### Preparation of the periodic mesoporous organosilicas (PMOs)

**Scheme 2:**
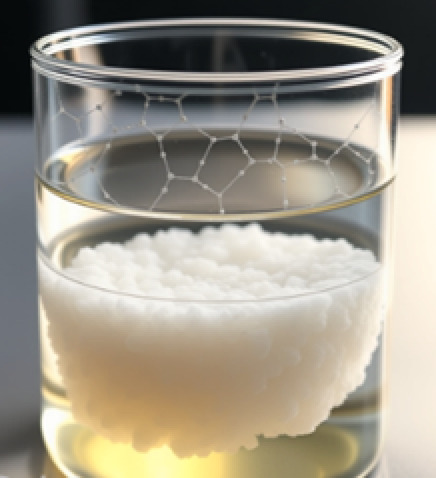
PMO framework diagram.

First, P123 (0.50 g), ethanol (3.50 mL), and deionized water (DW 1.10 g (0.5 mL)) were stirred at 20 °C. Then, TEOS (1.69 mL) was added and continuously stirred for 1 h at 20 °C. Next, BTPA (0.70 mL) was added drop by drop, followed by stirring for another hour. Finally, the mixture was washed three times with water and dried at 60 °C for 24 h (Scheme 2).

### Surfactant extraction of PMO

The surfactant was removed by soaking 0.250 g of the as-synthesized sample in 37.50 mL of EtOH in 0.850 mL of 36% HCl solution at 60 °C for 24 h. The resulting solid was recovered by filtration, washed with EtOH, and dried in an oven at 60 °C for 24 h. This material is referred to as the surfactant-extracted material.

### Preparation of the MXene (Ti_3_C_2_)

**Scheme 3:**
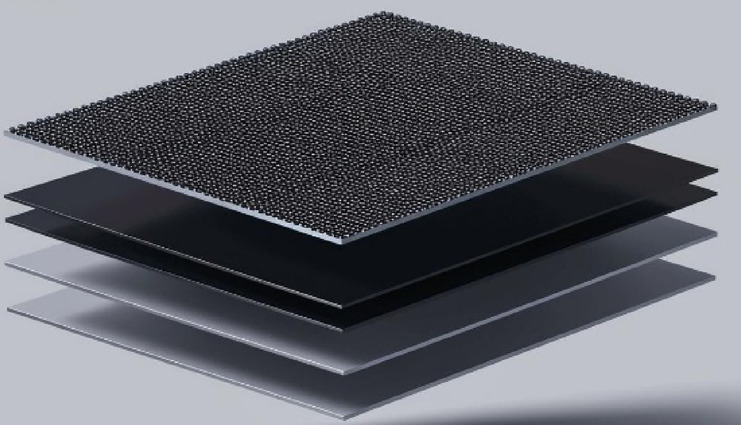
Structure of MXene.

Firstly, LiF (0.60 g) and HCl (15.0 mL) were stirred at room temperature for 30 min. Then, MAXphase (Ti_3_AlC_2_- 0.50 g) was slowly added to it. The resulting mixture was placed in a water bath for 72 h at 34 °C. The product is collected by a centrifuge, washed several times with HCl, EtOH, and water, and dried in a vacuum oven (4 h, 0.19 g, Scheme 3).

### Preparation of the amino-functionalized MXene sample

To start, 3-aminopropyl triethoxysilane (5.0 g) was added to a mixture of EtOH (18.0 g) and deionized water (2.0 g) and then vigorously stirred for 20 min. Following this, 0.20 g of precalculated MXene powder was immersed in the prepared solution. The mixture was transferred into a single-neck round-bottom flask and stirred at 80 °C for 24 h under reflux conditions. After completion, the resulting product was centrifuged using ethanol and deionized water to eliminate any residual modifier. Finally, the obtained product was dried in an oven at 75 °C for 24 h to achieve the amino-functionalized MXene sample (0.185 g).

### Preparation of the PMO@MXene nanocomposite

**Scheme 4:**
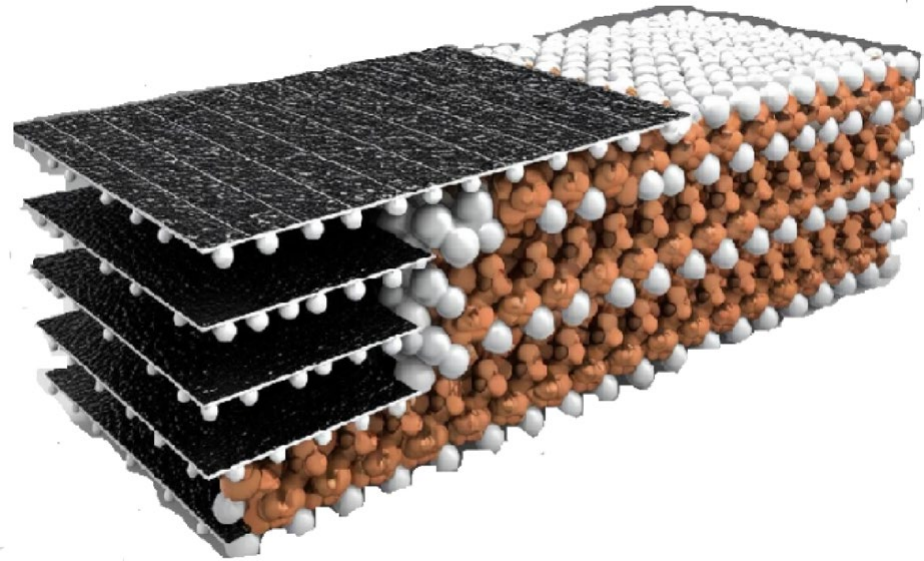
The structure of the PMO@MXene nanocomposite.

The physical synthesis method is employed for the preparation of the PMO@MXene nanocomposite. In this process, the amino-functionalized MXene sample (0.010 g) and PMO (0.0030 g) are stirred with water (1.10 g) and EtOH (3.50 mL) for 24 h at 60 °C. Subsequently, the obtained solid was separated and washed several times in a mixture of water and EtOH and dried for 24 h at 75 °C (0.013 g, Scheme 4).

### General procedure for the synthesis of 2-(aryl)-1 H-benzo[d]imidazole in the presence of the PMO@MXene nanocomposite

In a 5.0 mL reaction flask, a mixture of aldehyde (1a-l, 1.0 mmol), 1,2-phenylenediamine (2, 1.0 mmol), and the prepared catalyst (5.0 mg) in EtOH (2.0 mL) was heated under reflux for the appropriate time. The reaction progress was monitored by thin-layer chromatography (TLC- *n*-hexane: ethyl acetate (1:3)). After completion, the obtained solid, consisting of a mixture of the desired product and the catalyst, was isolated using a filter paper. The solubility of benzimidazole in ethyl acetate (EtOAc) was exploited to separate the PMO@MXene nanocomposite from the desired products. Finally, the obtained benzimidazole derivatives were crystallized from EtOAc with high purity.

## Results and discussion

Characterization of the nanoparticles typically involves various techniques to understand their size, shape, composition, surface properties, and other relevant characteristics. To confirm the structure and surface characteristics of the prepared PMO, MXene, and the PMO@MXene nanocomposite, several analyses were employed, including Fourier transform infrared spectroscopy (FT-IR) for identifying functional groups through vibrational modes, field-emission scanning electron microscopy (FESEM) for detailed imaging to reveal information about size and morphology, energy-dispersive X-ray spectroscopy (EDS) for determining the elemental composition of the nanoparticles, X-ray powder diffraction (XRD) for determining the crystallinity degree of the structure and providing insights into composition and arrangement, and High-resolution transmission electron microscopy (HRTEM) for imaging mode of specialized transmission electron microscopes that allows for direct imaging of the atomic structure of samples.

### Fourier-Transform infrared spectroscopy (FT-IR)

The FT-IR spectrum of the prepared PMO nanoparticles demonstrates apparent characteristics spanning the spectral range of 400–4000 cm⁻¹. The observed bands in this spectrum can be specified as follows: 1470 cm^− 1^; Relates to the bending vibrations of the Si-O-Si bonds; 1794 cm^− 1^; confirms the asymmetric stretching vibrations of Si-O-Si; 940 cm^− 1^; shows the Si-OH stretching vibrations or Si-O-Si symmetric stretching vibrations; 1360 cm^− 1^; can be related to the CH_3_ shaking vibrations or Si-CH_3_ stretching vibrations. 1472 cm^− 1^; relates to the CH_2_ bending vibrations; 2820 cm^− 1^; is related to the symmetric stretching vibrations of CH_2_; 2943 cm^− 1^; clarifies the symmetric stretching vibrations of CH_3_; and 3431 cm^− 1^; can be a result of the O-H stretching or N-H stretching vibrations.

The FTIR spectrum provides valuable insights into the functional groups present in the MXene structure. The broad peak around 3400 cm⁻¹ corresponds to the stretching vibrations of hydroxyl groups on the MXene surface. The presence of these hydroxyl groups indicates that the MXene surface is partially oxidized or has undergone surface modification. Additionally, the peak near 1600 cm⁻¹ is attributed to the stretching vibrations of the metal-carbon (M–C) bonds, highlighting the characteristic MXene composition, which typically includes a transition metal (such as Al) and carbon (C). Furthermore, the absorption peak around 1100 cm⁻¹ corresponds to the bending vibrations of the Ti–O bonds. This observation suggests that the MXene surface may be partially oxidized, leading to the formation of metal-oxygen (M–O) functional groups.

**Fig. 1:**
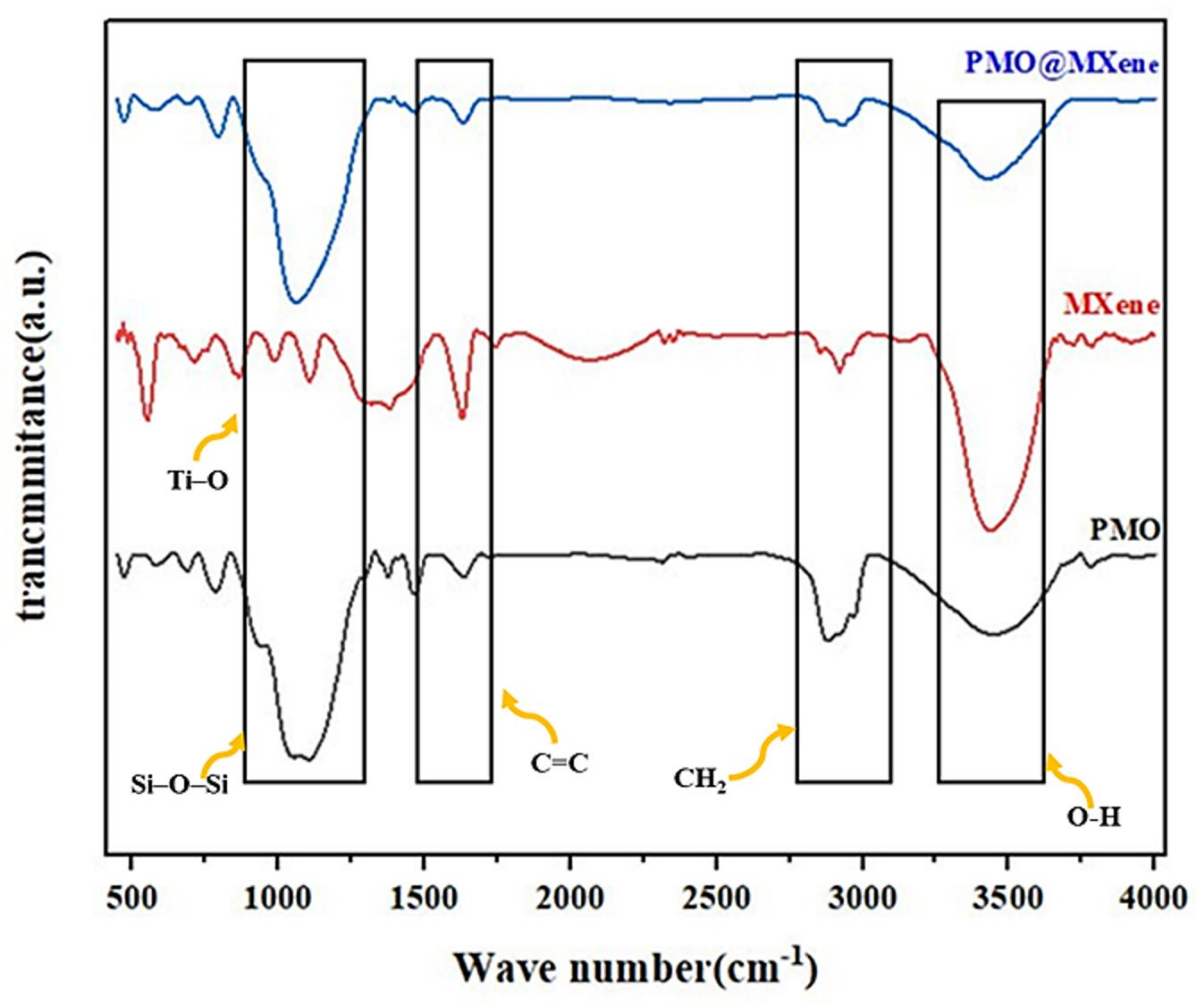
FT-IR of PMO, MXene, and the PMO@MXene nanocomposite.

The FTIR spectrum reveals several significant absorption bands, providing insight into the chemical structure and functional groups of the PMO@MXene nanocomposite. In the range of 1000–1200 cm⁻¹, a strong absorption band corresponds to the symmetric and asymmetric stretching vibrations of the Si–O–Si bonds, characteristic of silica frameworks, particularly PMOs, thereby confirming their presence in the nanocomposite. Peaks in the region of 500–800 cm⁻¹ indicate bending vibrations of the functional groups associated with the MXene materials, which may include M–O, M–C, or Ti–O bonds, depending on the specific composition of the MXene. Broad absorption bands in the 3000–3500 cm⁻¹ region are attributed to the O–H stretching vibrations, likely arising from hydroxyl groups on the surface or adsorbed water molecules, which highlights the hydrophilic nature of the nanocomposite. Furthermore, the peak at 1600 cm⁻¹ may correspond to the C = C stretching vibrations, suggesting the presence of organic moieties. The peak could also be a result of the H–O–H bending vibrations of adsorbed water molecules (Fig. 1).

### Field-emission scanning electron microscopy (FESEM)

**Fig. 2:**
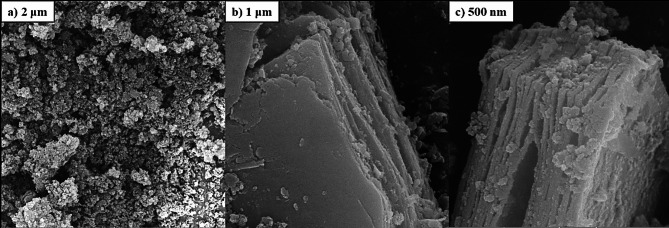
FESEM images of (**a**) PMO (2 μm), (**b**) MXene (1 μm), and (**c**) the PMO@MXene nanocomposite (500 nm).

The SEM images of the gels prepared with P123 are shown in Fig. 2a. These images indicate that the resulting gels possess a well-ordered mesostructure, reflecting the formation of a uniform and porous network at the microscopic level. Such a structure is highly important for catalytic applications and surface adsorption, as it provides a larger active surface area and enhances particle permeability. The SEM micrograph of the prepared PMO gel, as observed in Fig. 2a, shows a distinct mesostructured with clearly visible pores and regular channels. This mesostructure plays a crucial role in mechanical stability and the uniform distribution of active particles on the gel surface, serving as a suitable framework for the formation of nanocomposites.

Figure 2b displays the SEM image of the original MXene, illustrating its characteristic accordion-like structure. This layered and ordered structure is a notable feature of MXenes and, due to its large surface area and high ion-exchange capacity, is suitable for the formation of nanocomposites and catalytic applications.

As shown in Fig. 2c, the MXene layers are separated from each other, and the PMO particles, which form a physical nanocomposite with MXene, are located between and on the MXene layers. This arrangement not only helps stabilize the nanocomposite structure but also increases the contact area between PMO and MXene, enhancing mass transfer and catalytic interactions between the two components. Furthermore, such a layered arrangement ensures a uniform distribution of PMO particles, preventing aggregation and clumping, which is essential for optimal catalyst performance. Nanoartography, a method to make SEM images resembling the real world is used for Figs. S4a and S4b.

### Mapping elemental analysis of the PMO@MXene nanocomposite and EDSs of PMO, MXene, and the PMO@MXene nanocomposite

**Fig. 3:**
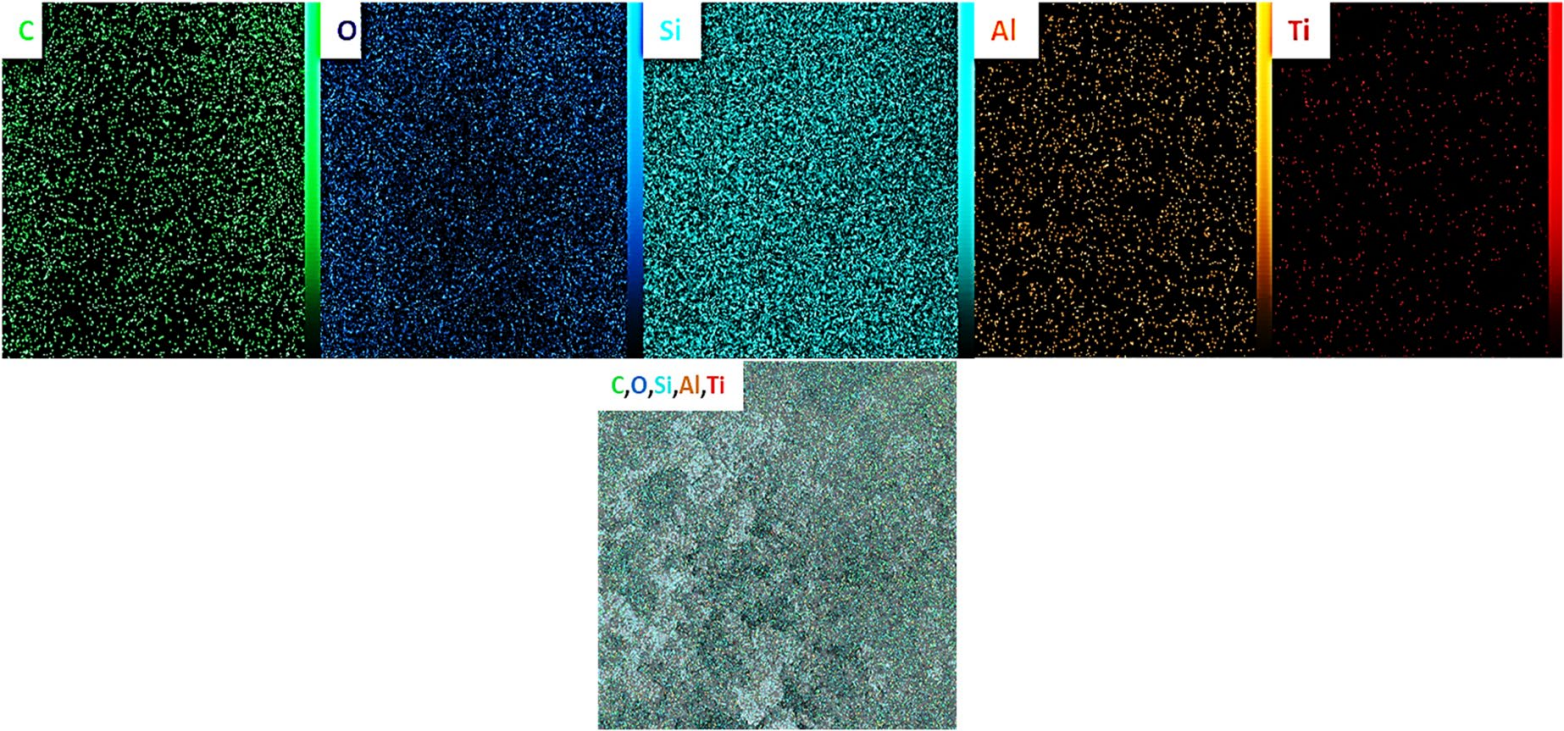
EDS elemental mapping analysis of the PMO@MXene nanocomposite.

**Fig. 4:**
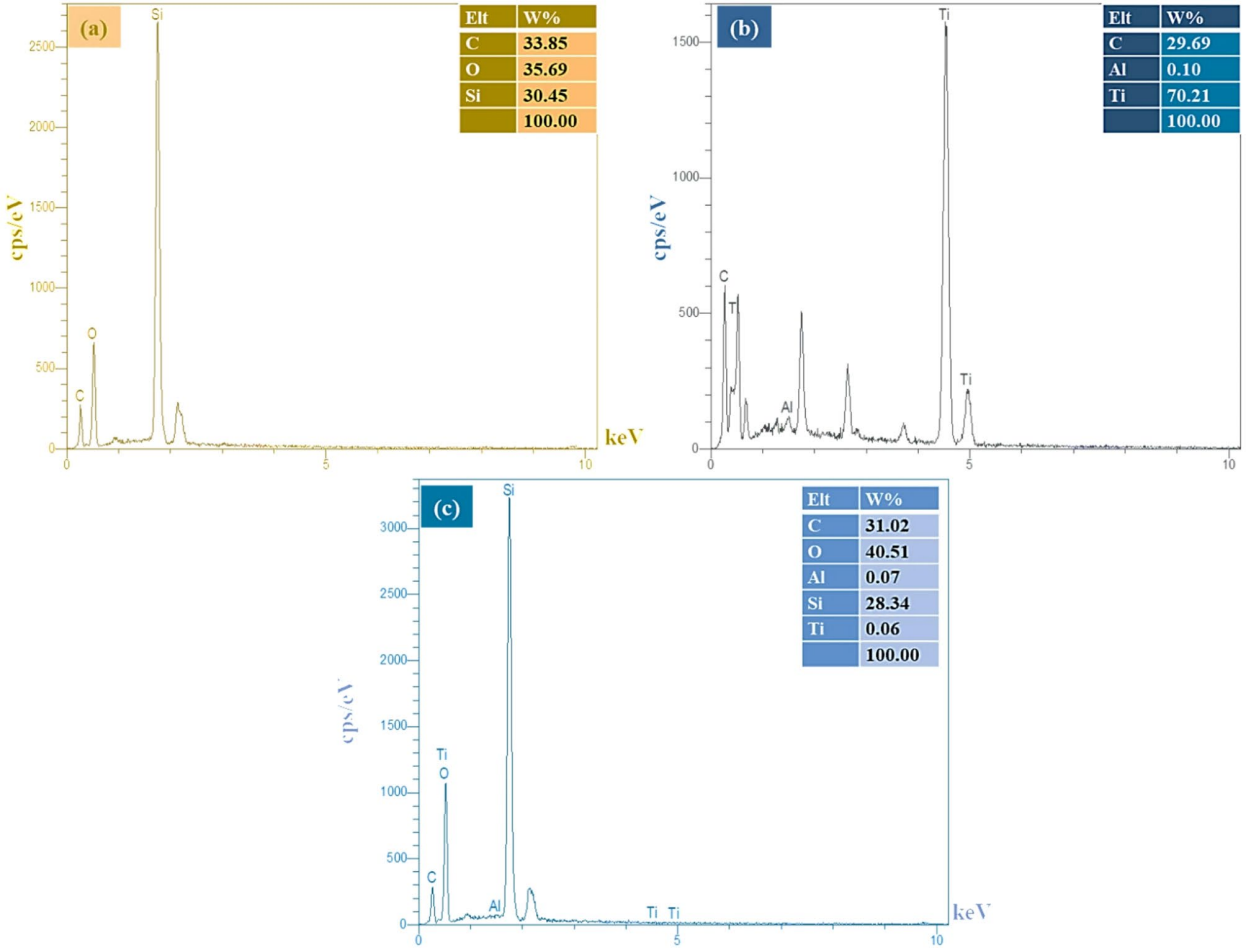
Elemental analysis by energy-dispersive X-ray spectroscopy (EDS) of (**a**) PMO, (**b**) MXene, and (**c**) the PMO@MXene nanocomposite.

As shown in Fig. 3, the elemental mapping analysis of the PMO@MXene catalyst confirms the existence of C, O, Si, Al, and Ti elements in the definite composition of the nanomaterial. In addition, the mapping elemental analysis demonstrates a uniform distribution of desired elements. Furthermore, the identification of C, O, and Si (Fig. 4a), C, Ti, and O (Fig. 4b), and C, Ti, O, and Si (Fig. 4c) elements in the EDS analysis serves as additional evidence supporting the successful preparation of the requested samples.

### Powder X-ray diffraction (XRD) of PMO, MXene, and the PMO@MXene nanocomposite

**Fig. 5:**
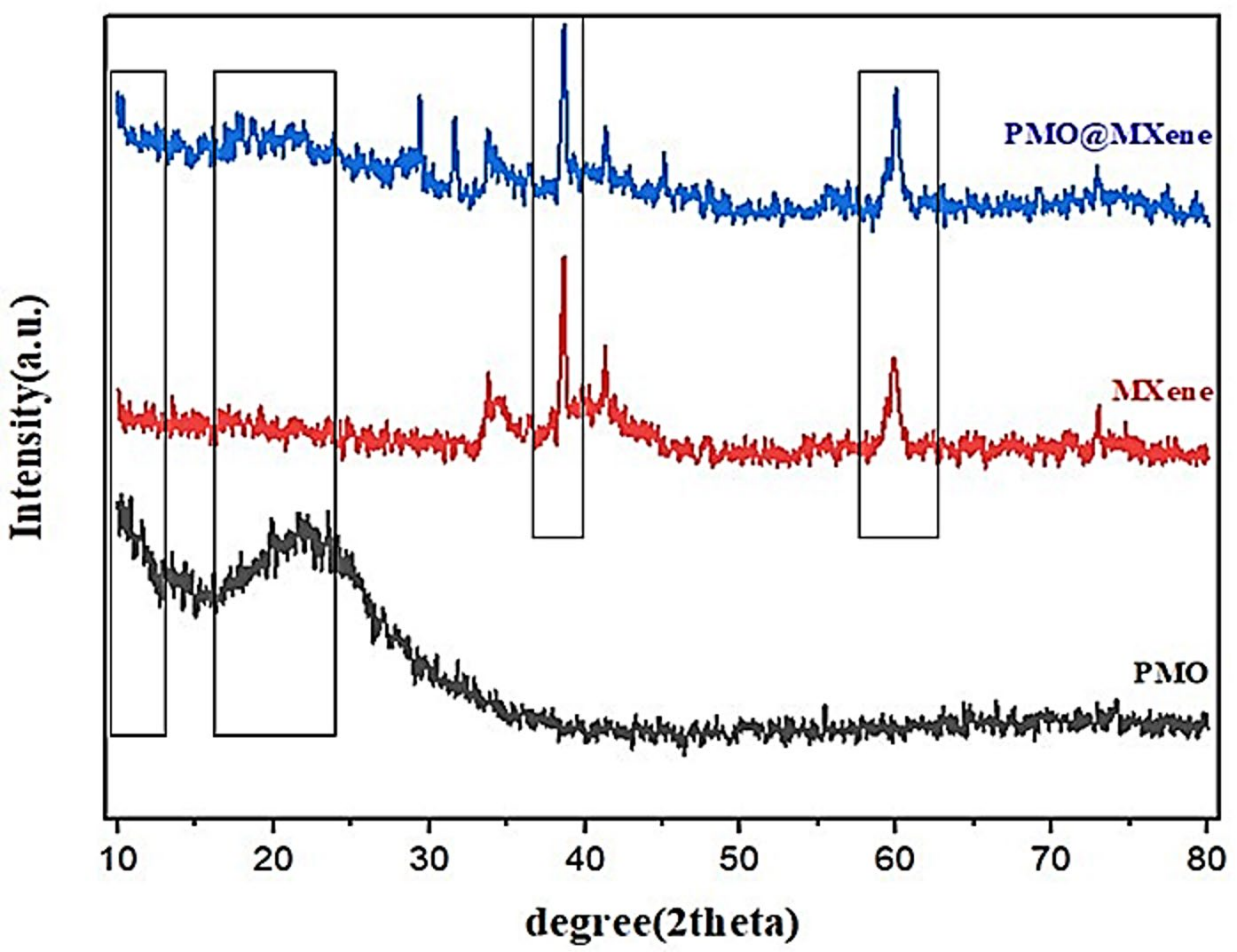
X-ray diffraction patterns of PMO, MXene, and the PMO@MXene nanocomposite.

The X-ray diffraction (XRD) patterns illustrate how various processes, such as etching, exfoliation, and aminosilane modification, affect the crystalline structure of MXene. The addition of Ti₃AlC₂ powder to a mixture of hydrochloric acid and lithium fluoride initiates a reaction that produces hydrogen gas bubbles. This reaction results in the disappearance of several peaks in the 34–45° range and a shift of the (002) and (004) peaks associated with the MAX phase to lower angles. These changes indicate the effective removal of the aluminum layer in the MXene structure. XRD analysis of PMO reveals a single broad peak in the low-angle diffraction range (between 1° and 5°) at approximately 2θ = 1.11°. This low-angle region (~ 1–3°) corresponds to the well-ordered mesoporous structure, confirming the presence of long-range mesostructural order due to the periodic arrangement of the silica walls. A broad peak in the 15–30° range is characteristic of amorphous silica (SiO_2_). PMO materials are predominantly amorphous, consisting of silica frameworks with organic bridges, which leads to the absence of distinct crystalline reflections. The combination of the sharp MXene peak with broad secondary phase peaks and amorphous halos provides evidence of the successful integration of the PMO and MXene components in the composite. The structural information obtained from this XRD pattern enhances the understanding of the composition, morphology, and potential applications of the PMO@MXene system. A sharp peak at 2θ = 6.5° corresponds to the (002) reflection of the MAX phase, indicating an interlayer spacing of approximately 13.6 Å between the MXene layers. The high intensity of this peak suggests a significant amount of well-ordered crystalline MXene phase within the nanocomposite. Broad peaks in the 2θ = 20–30° range can be attributed to the presence of secondary phases such as TiC and Al_2_O_3_. The broad nature of these peaks suggests that these secondary phases are likely nanocrystalline or have a disordered structure. The positions of these peaks correspond to the (111), (200), and (220) reflections of the TiC phase, as well as the (012), (104), and (110) reflections of the Al_2_O_3_ phase. Furthermore, broad peaks in the 2θ = 40–60° range confirm the presence of amorphous or nanostructured components in the prepared nanocomposite, indicating the integration of the mesoporous silica framework within the MXene structure. These features suggest that the mesoporous phase exhibits a disordered and non-crystalline structure within the composite (Fig. 5).

Employing Scherer’s equation for particle size of the pristine PMO, MXene, and PMO@MXene nanocomposite calculation at 2θ = 10.83^o^, 2θ = 38.63^o^, and 2θ = 38.66^o^ and a full width at half maximum (FWHM) of 117.71, 0.32, and 0.313 shows a determined particle size of 11.84, 13.5, and 5.1 nm and the Brunauer-Emmett–Teller (BET) analysis revealed a specific surface area of 92.8 m²/g for the heterogeneous PMO@MXene nanocomposite (Fig. S15).

### HRTEM and SEAD of the samples

**Fig. 6:**
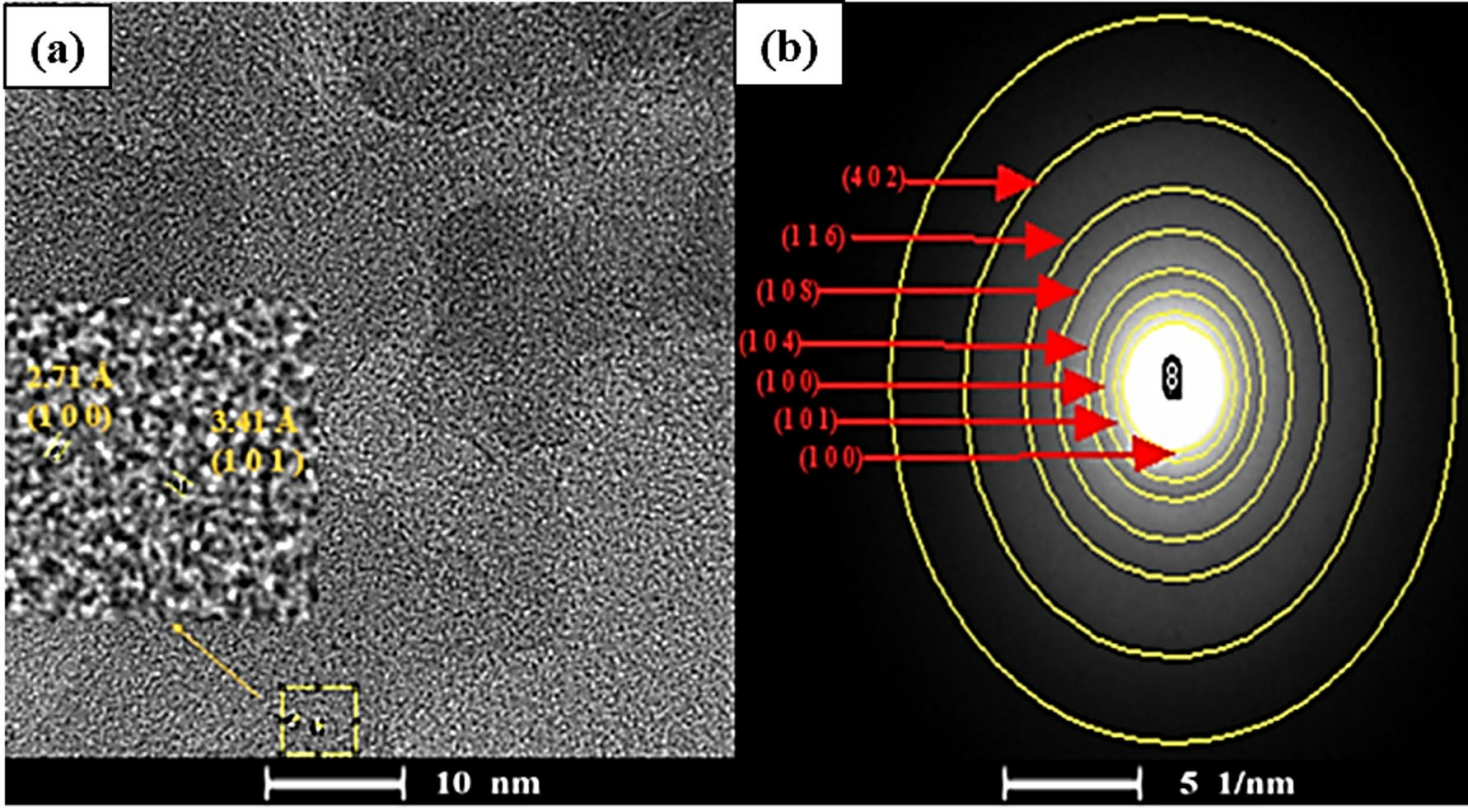
(**a**) HRTEM analysis and (**b**) SAED analysis of the PMO@MXene nanocomposite.

As illustrated in Fig. 6, the obtained Miller indices (1 0 0) bright spots, (1 0 1), and (4 0 2), for PMO (reference code: 00-033-1161) and (1 0 0), (1 0 4), (1 0 8), and (1 1 6) for Mxene (reference code: 96-722-1325) from the SAED analysis, along with d-spacing values of 3.41 Å and 2.71 Å determined through HRTEM analysis for the Miller indices (1 0 1) reference code: 00-033-1161) and (1 0 0) (reference code: 96-722-1325) respectively for PMO and MXene, indicate the successful synthesis of PMO@MXene.

### Systematic exploration of optimal conditions for the synthesis of benzimidazole derivatives

After the identification of the prepared nanocomposite and to study of its promoting ability, the efficiency of this reagent was investigated in the synthesis of benzimidazole derivatives. Firstly, to explore the optimal synthesis of the mentioned target molecules, a model reaction involving 2-chlorobenzaldehyde (1a), 1,2-phenylenediamine (2), and PMO@MXene as the catalyst was selected and the effect of various reaction parameters, including solvent type, catalyst loading, and temperature, were systematically investigated on it (Table 1). Initially, the reaction was performed without a catalyst, both at room temperature and under reflux, using water as the solvent (Table 1, entry 1). The results demonstrated that no product formation was observed under these conditions, emphasizing the necessity of a catalytic system for effective conversion. Subsequently, the catalytic performance of different catalysts, including PMO, MXene, and PMO@MXene, was studied in water at room temperature (Table 1, entries 2–4). Among these, PMO@MXene displayed superior activity, achieving a yield of 25% (Entry 4) compared to PMO and MXene alone. After identifying the PMO@MXene as the efficient catalyst, the effect of different solvents, such as MeOH, EtOH, EtOAc, CH_2_Cl_2_, and the mixture of H_2_O and EtOH, on the reaction yield was examined (Table 1, entries 5–9). The results revealed that ethanol (EtOH) provided the best performance, with a yield of 38% under room temperature conditions (Table 1, entry 6). The catalyst loading was then optimized, demonstrating that increasing the PMO@MXene quantity from 2.0 mg to 5.0 mg significantly enhances the reaction yield, reaching to 98% in just 10 min under reflux conditions in ethanol (Table 1, entry 12). Further increasing the catalyst loading to 7.50 mg (Table 1, entry 13) slightly decreased the yield to 92%, likely due to the catalyst oversaturation. Temperature effects were also investigated, showing that raising the reaction temperature from room temperature to reflux drastically improved the yield (Table 1, entries 10–12). Overall, the optimal conditions were determined to be 5.0 mg of PMO@MXene as the catalyst in ethanol under reflux, resulting in excellent yields and reduced reaction times.

**Table 1:** Exploring optimized conditions for the one-pot synthesis of 2-(aryl)-1-*H*-benzo[d]imidazole derivatives obtained from the reaction of 2-chlorobenzaldehyde (**3a**) and 1,2-phenylene Diamine (**2**).

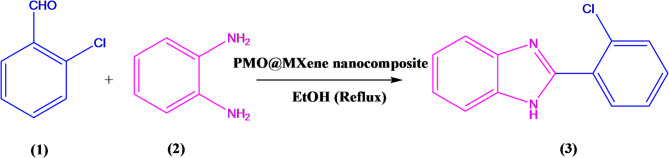					
Entry	Catalyst loading (mg)	Solvent (mL)	Temperature (°C)	Time (min)	Yield^a, b^ (%)
1	–	H_2_O (2.0)	r.t	45	–
2	PMO (2.0)	H_2_O (2.0)	r.t	45	15
3	MXene (2.0)	H_2_O (2.0)	r.t	60	23
4	PMO@MXene (2.0)	H_2_O (2.0)	r.t	40	25
5	PMO@MXene (2.0)	MeOH (2.0)	r.t	45	28
6	PMO@MXene (2.0)	EtOH (2.0)	r.t	45	38
7	PMO@MXene (2.0)	EtOAc (2.0)	r.t	45	26
8	PMO@MXene (2.0)	CH_2_Cl_2_ (2.0)	r.t	45	32
9	PMO@MXene (2.0)	H_2_O/EtOH (2.0)	r.t	45	30
10	PMO@MXene (2.0)	–	r.t	45	57
11	PMO@MXene (2.0)	–	80	30	68
**12**	**PMO@MXene (5.0)**	**EtOH (2.0)**	**80**	**10**	**98**
13	PMO@MXene(7.5)	–	80	15	92
14	PMO@MXene (5.0)	–	80	15	31
15	–	–	80	15	-

^a^Reaction conditions: aldehyde (**1a**, 1.0 mmol), 1,2-phenylene diamine (**2**, 1.0 mmol), and 2.0 mL EtOH as a solvent. ^b^The yield refers to the isolated pure product **3a**.

Table 2 outlines the efficient synthesis of 2-(aryl)-1*H*-benzimidazole derivatives using the PMO@MXene as a heterogeneous catalyst under the optimized conditions. The reaction involves the condensation of a variety of aromatic aldehydes with 1,2-phenylenediamine. The aldehydes tested feature various substituents, including electron-withdrawing groups (e.g., -NO_2_, -Cl, -Br) and electron-donating groups (e.g., -OMe, -OH), demonstrating the catalyst’s broad applicability. The methodology consistently achieves high yields (87%–98%) during short reaction times (10–20 min), underscoring the catalyst’s remarkable activity.

The study highlights that aldehydes with electron-withdrawing substituents tend to react more rapidly due to enhanced carbonyl electrophilicity, whereas aldehydes containing electron-donating substituents and heterocyclic aldehydes require longer reaction times due to their reduced reactivity. This trend aligns with the reaction mechanism, wherein nucleophilic attack on the aldehyde carbonyl group constitutes the rate-determining step. The use of PMO@MXene not only accelerates the reaction but also provides a green, reusable catalytic system, making it a promising approach for synthesizing bioactive benzimidazole derivatives with the potential of pharmaceutical applications.

**Table 2:**
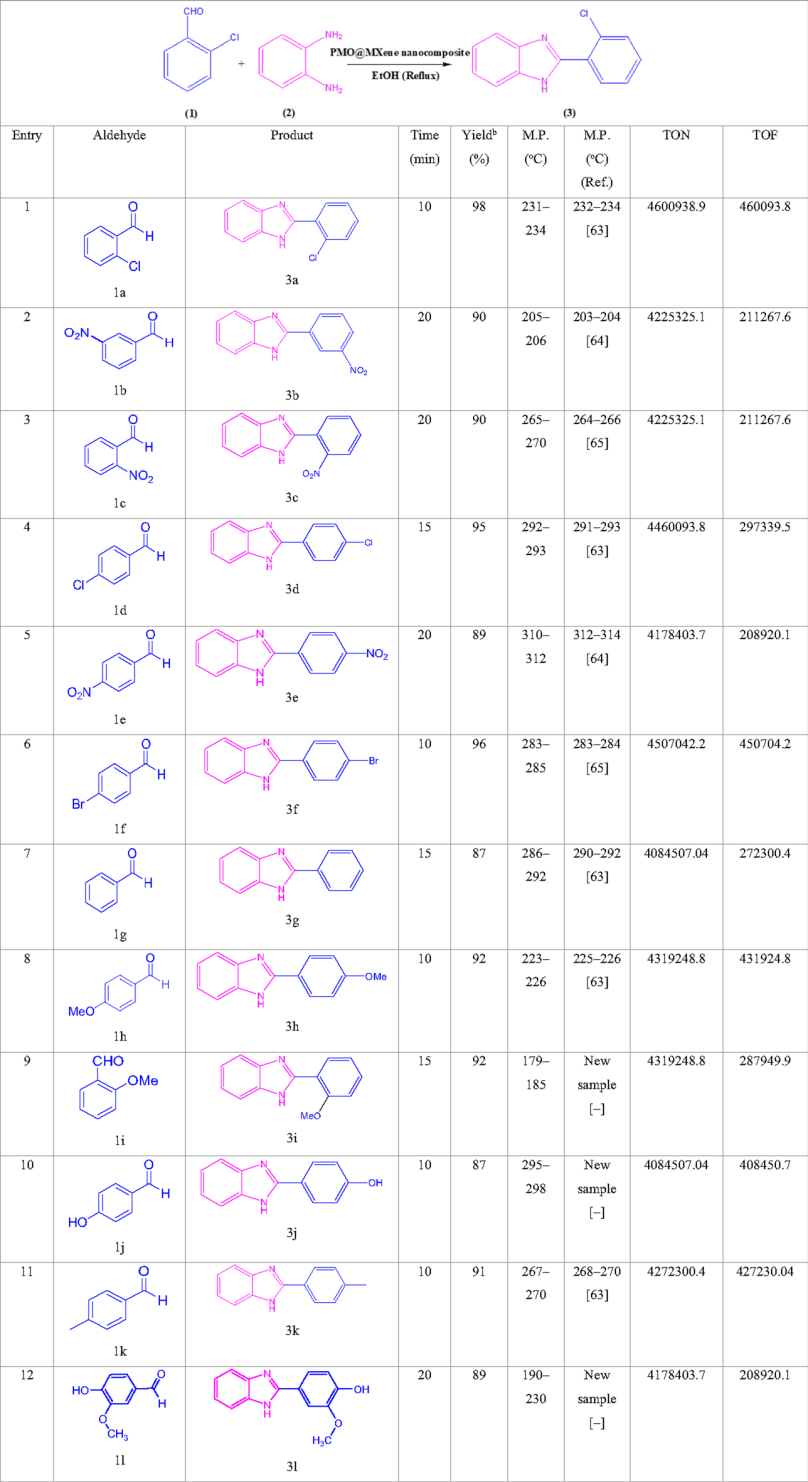
Synthesis of diverse 2-(aryl)-1*H*-benzimidazole (3a-l) using various aldehydes in the presence of the PMO@MXene nanocomposite.

Reaction conditions: aromatic aldehyde (1a–l, 1.0 mmol), 1,2-phenylene diamine (2, 1.0 mmol), PMO@MXene (5.0 mg), and EtOH (2.0 mL) under reflux (80 °C) conditions.

^a^The yields refer to the isolated pure products.

### Proposed mechanism for the synthesis 2-(aryl)-1 H-benzimidazole derivatives 3a-l catalyzed by the PMO@MXene nanocomposite

**Fig. 7:**
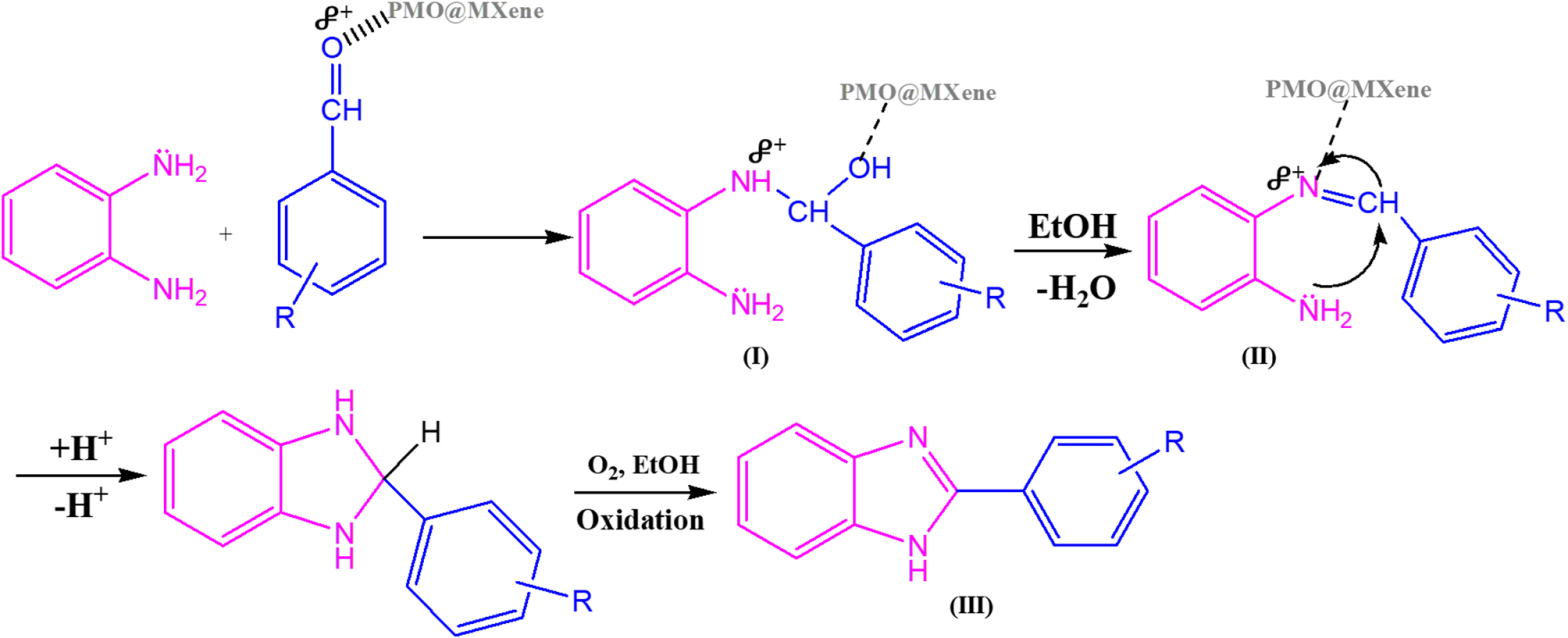
Proposed mechanism for the synthesis of 2-(aryl)-1*H*-benzo[*d*]imidazole derivatives in the presence of the PMO@MXene nanocatalyst.

Figure 7 presents the proposed mechanism for the PMO@MXene-catalyzed synthesis of 2-(aryl)-1*H*-benzimidazoles (3a–l). Surface acidic sites of the PMO@MXene coordinate to the aldehyde C = O, increasing electrophilicity of the carbonyl carbon and facilitating nucleophilic attack by the primary amine to give a tetrahedral intermediate (I). Subsequent catalyst-assisted dehydration yields an imine intermediate (II). Intramolecular nucleophilic attack on the imine, promoted by the catalyst, closes the ring; final oxidation (air/O₂) furnishes the benzimidazole core. This pathway is supported by in-situ FT-IR (disappearance of the C = O band and appearance of C = N), time-resolved ¹H NMR aliquots, control experiments with PMO and MXene alone, and reduced conversion under an inert atmosphere, collectively indicating the cooperative acid-activation and oxidation roles of the PMO@MXene^66,67^.

### Investigating the recyclability and reusability of the PMO@MXene nanocomposite

**Fig. 8:**
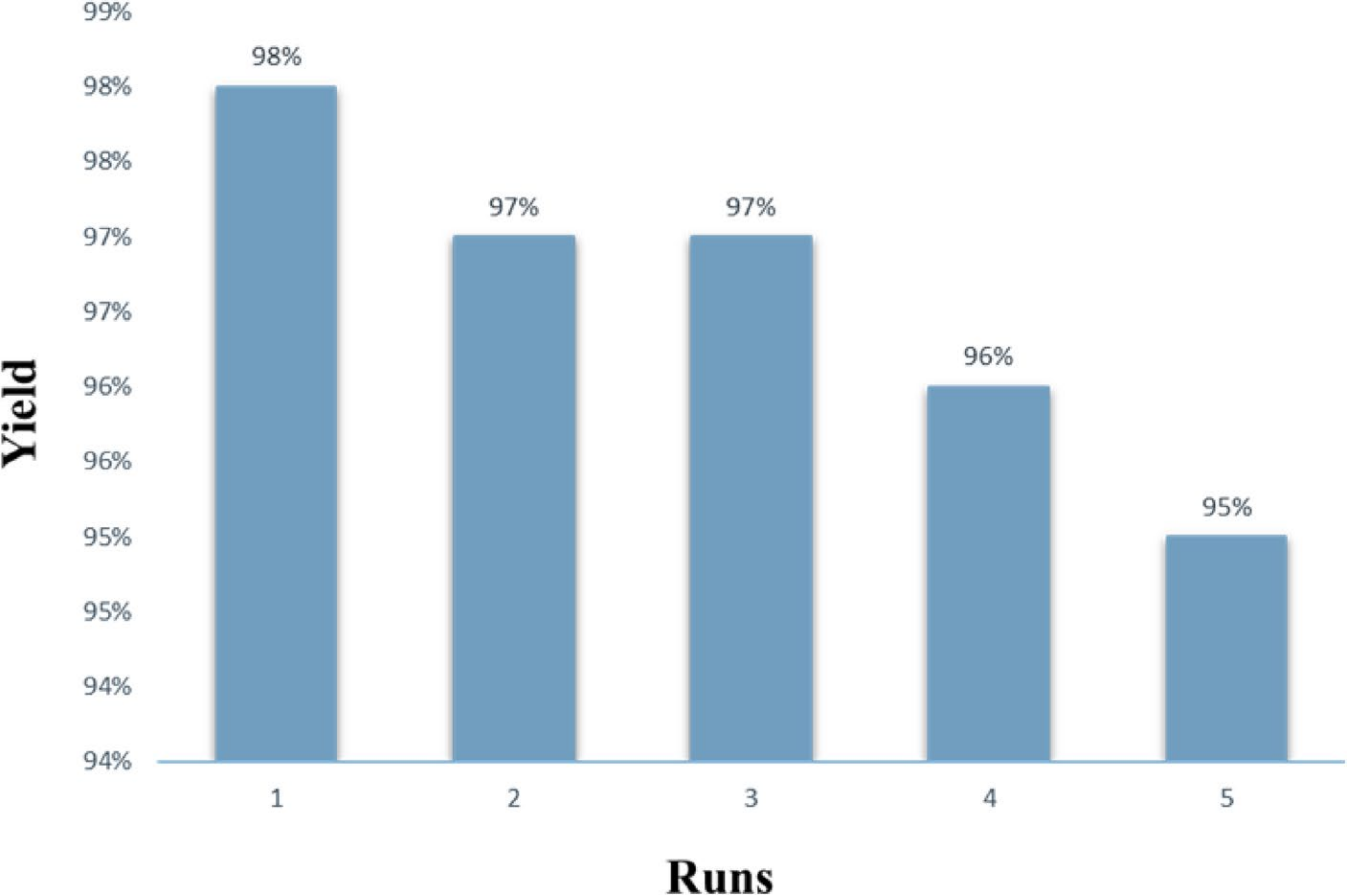
The results of recyclability and reusability of the PMO@MXene nanocomposite in the model reaction.

The recyclability of the PMO@MXene as an important feature, which shows its compatibility with the green chemistry rules, was evaluated. Following each reaction cycle, the catalyst was separated from the reaction mixture using filter paper and thoroughly washed with EtOAc and EtOH to remove any residual impurities. It was then dried at 65 °C for 2 h before being reused in the same model reaction. As shown in Fig. 8, the recycled catalyst maintained high conversion rates over multiple reaction cycles, demonstrating its excellent catalytic efficiency. Even after five consecutive cycles, the catalyst achieved an average yield of 96.7%, underscoring its stability and reusability. Notably, the catalyst’s structural integrity remained largely intact throughout the reuse process, further confirming its durability. These findings confirm the strong potential of PMO@MXene as a robust and efficient heterogeneous nanocomposite for the three-component synthesis of 2-(aryl)-1*H*-benzo[d]imidazole derivatives. Its sustained performance over multiple cycles suggests that it can be reused without significant loss of activity, making it a promising candidate for catalytic applications under optimal reaction conditions (Fig. 8).

**Fig. 9:**
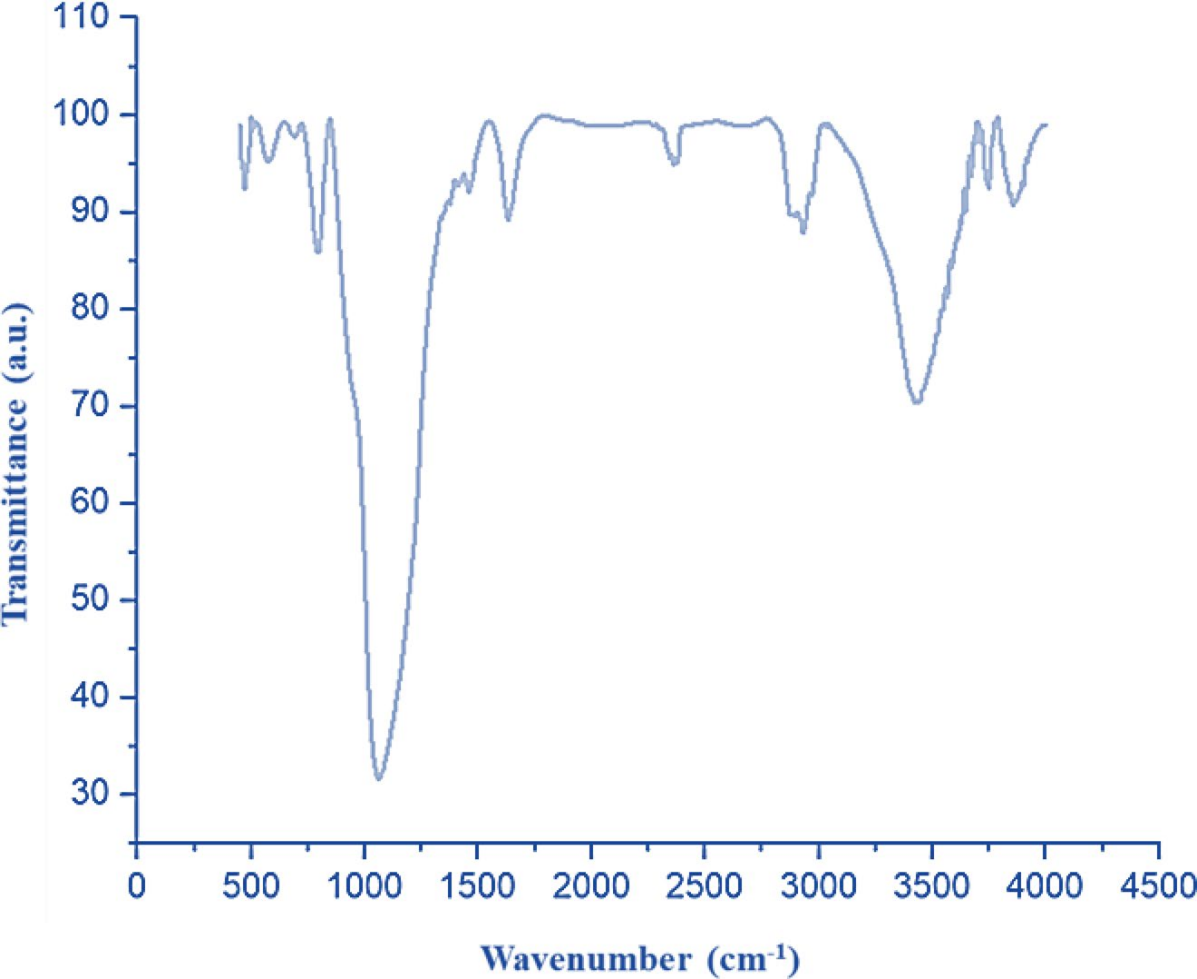
FT-IR spectrum of the recycled PMO@MXene nanocomposite after 5 runs.

As shown in Fig. 9, the FT-IR of the nanocomposite doesn’t show any change in the structure of the PMO@MXene nanocatalyst after 5 runs.

### Comparison of different protocols for the synthesis of 2-(aryl)-1 H-benzimidazoles

The effectiveness of the PMO@MXene catalyst was evaluated in comparison with the previously established protocols. As detailed in Table 3, the PMO@MXenes nanocatalyst demonstrates remarkable efficiency in the swift synthesis of 2-(aryl)-1*H*-benzimidazoles. A distinguishing feature of this catalyst is its ability to promote the formation of 2-(aryl)-1*H*-benzimidazole products while adhering to environmentally friendly practices and principles of green chemistry.

**Table 3:** Comparison of the effectiveness of PMO@MXene in the promotion of the synthesis of 2-(aryl)-1*H*-benzo[*d*]imidazoles with some of the recently reported protocols.

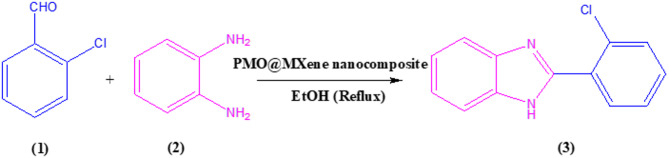					
Entry	Catalyst loading	Reaction conditions	Time (min.)	Yield (%)	Ref.
**1**	In(OTF)_3_/ 5.0 mol%	r.t/Solvent-free	30	95	^68^
**2**	Na_3_AlF_6_/ 2.0 mol%	50 °C/ EtOH	120	80	^69^
**3**	LaCl_3_/ 10.0 mol%	r.t/ CH_3_CN	120	95	^70^
**4**	Dioxane dibromide 100.0 mol%	r.t/ CH_3_CN	30–60	87	^71^
**5**	Zn (OTf)_2_ 10.0 mol%	Reflux/ EtOH	480	95	^72^
**6**	PMO@MXene 5.0 mg	80 °C/ EtOH	10	98	This work

## Conclusion

In summary, we have successfully developed and applied a novel PMO@MXene nanocomposite as an efficient, recyclable, and environmentally friendly catalyst for the synthesis of 2-(aryl)-1*H*-benzo[*d*]imidazole derivatives for the first time. The strategic integration of periodic mesoporous organosilica with conductive MXene layers resulted in a hybrid system that benefits from enhanced surface area, stability, and catalytic efficiency. The PMO@MXene nanocatalyst demonstrated outstanding performance under mild and green reaction conditions, achieving excellent yields and selectivity with minimal catalyst loading. Furthermore, its remarkable recyclability highlights its potential for long-term and cost-effective use in organic synthesis. Given its unique structural features, superior catalytic activity, and alignment with green chemistry principles, the PMO@MXene system opens new avenues for the design of advanced heterogeneous catalysts for sustainable heterocyclic synthesis and other value-added chemical transformations.

## Supplementary Information

Below is the link to the electronic supplementary material.

Supplementary Material 1

## Data Availability

Data availability is detailed within the article and its Supplementary Information. Source data are also provided alongside the publication.
