# Supplementary material for: Heterogeneous PMO@MXene nanocomposite: a robust and efficient nanocatalyst for two-component synthesis of highly substituted 2-(aryl)-1H-benzo[d]imidazole derivatives
Source: Sci Rep. 2025 Nov 28;15:44560. doi: 10.1038/s41598-025-28219-8 (PMC12738787; doi:10.1038/s41598-025-28219-8)
Supplement: Supplementary file 1 — Supplementary Material 1 [file 41598_2025_28219_MOESM1_ESM.docx]

**Supporting Information for**

**Heterogeneous PMO@MXene nanocomposite: A robust and efficient nanocatalyst for two-component synthesis of highly substituted 2-(aryl)-1*H*-benzo[d]imidazole derivatives**

Safa Hnaifi,^a^ Farhad Shirini,^a*^ Bahram Ramezanzade,^b^ Hassan Tajik^a^

*^a^* *Department of Organic Chemistry, Faculty of Chemistry, University of Guilan, Rasht, 41335-19141, Iran*

*^b^* *Department of Surface Coating and Corrosion, Institute for Color Science and Technology, Tehran, Iran*

*Corresponding author: [shirini@guilan.ac.ir](mailto:shirini@guilan.ac.ir), [fshirini@gmail.com](mailto:fshirini@gmail.com)

| **Contents** | **Page** |
| --- | --- |
| Title page | 1 |
| Materials and Methods | 4 |
| Characterization | 4 |
| Fig S1: General scheme of the PMO@MXene nanocatalyst synthesis and its use in the synthesis of 2(aryl)-1*H*-benzo[d]imidazole derivatives (Graphical abstract). | 4 |
| Scheme S1: Commercial drugs containing benzimidazole ring. | 5 |
| Scheme S2: PMO framework diagram. | 5 |
| Scheme S3: Structure of MXene. | 5 |
| Scheme S4: The structure of the PMO@MXene nanocomposite. | 6 |
| Fig S2: FT-IR of PMO, MXene, and the PMO@MXene nanocomposite. | 6 |
| Fig S3: FESEM images of a) PMO (2 μm), b) MXene (1 μm), and c) the PMO@MXene nanocomposite (500 nm). | 7 |
| Fig S4: FESEM image of (a) MXene like a snowy mountain and (b) PMO like a jungle designed by the Nanoart method. | 7 |
| Fig. S5: FESEM image of the recycled PMO@MXene nanocomposite (1 μm). | 8 |
| Fig S6: EDS elemental mapping analysis of the PMO@MXene nanocomposite. | 8 |
| Fig S7: Elemental analysis by energy-dispersive X-ray spectroscopy (EDS) of a) PMO, b) MXene, and c) the PMO@MXene nanocomposite. | 8 |
| Fig S8: X-ray diffraction patterns of PMO, MXene, and the PMO@MXene nanocomposite. | 9 |
| Fig S9: HRTEM analysis (a), and SAED analysis (b) of the PMO@MXene nanocomposite. | 9 |
| Fig. S10: The TEM images of the (a) PMO@MXene and (b) recycled PMO@MXene nanocomposite | 10 |
| Table S1: Exploring optimized conditions for the one-pot synthesis of 2-(aryl)-1*H*benzo[d]imidazole derivatives obtained from the reaction of 2-chloro benzaldehyde (3e) and 1,2phenylene diamine (2). | 12 |
| Table S2: Synthesis of diverse 2-(aryl)-1*H*-benzo[*d*]imidazole (3a-l) using various aldehydes in the presence of the PMO@MXene nanocomposite.a | 11 |
| Fig. S11: Proposed mechanism for the synthesis of 2-(aryl)-1*H*-benzimidazole derivatives in the presence of the PMO@MXene nanocatalyst. | 14 |
| Fig. S12: The results of recyclability and reusability of the PMO@MXene nanocomposite in the model reaction. | 14 |
| Fig. S13: FT-IR spectrum of the recycled PMO@MXene nanocomposite after 5 runs. | 15 |
| Fig. S14: X-ray diffraction patterns of the recycled PMO@MXene nanocomposite. | 15 |
| Table S3: Comparison of the effectiveness of PMO@MXene in the promotion of the synthesis of 2-(aryl)-1*H*-benzo[*d*]imidazole with some of the recently reported protocols. | 16 |
| Fig. S15: Brunaur-Emmet-Teller (BET) surface area analysis of the PMO@MXene nanocomposite. | 17 |
| Fig. S16: ^1^HNMR of the 2-(2-chlorophenyl)-1*H*-benzo[*d*]imidazole. | 18 |
| Fig. S17: ^1^HNMR of the 2-(3-nitrophenyl)-1*H*-benzo[*d*]imidazole. | 19 |
| Fig. S18: ^1^HNMR of the 2-(2-nitrophenyl)-1*H*-benzo[*d*]imidazole. | 20 |
| Fig. S19: ^1^HNMR of the 2-(4-chlorophenyl)-1*H*-benzo[*d*]imidazole. | 21 |
| Fig. S20: ^1^HNMR of the 2-(4-nitrophenyl)-1*H*-benzo[*d*]imidazole. | 22 |
| Fig. S21: ^1^HNMR of the 2-(4-bromophenyl)-1*H*-benzo[*d*]imidazole. | 23 |
| Fig. S22: ^1^HNMR of the 2-phenyl-1*H*-benzo[*d*]imidazole. | 24 |
| Fig. S23: ^1^HNMR of the 2-(4-methoxyphenyl)-1*H*-benzo[*d*]imidazole. | 25 |
| Fig. S24: ^1^HNMR of the 2-(2-methoxyphenyl)-1*H*-benzo[*d*]imidazole. | 26 |
| Fig. S25: ^1^HNMR of the 2-(4-hydroxyphenyl)-1*H*-benzo[*d*]imidazole. | 27 |
| Fig. S26: ^1^HNMR of the 2-phenyl-1*H*-benzo[*d*]imidazole. | 28 |
| Fig. S27: ^1^HNMR of the 2-(1*H*-benzo[*d*]imidazole-2-yl) methoxyphenpl). | 29 |
| Fig. S28: ^13^CNMR of the 2-(2-methoxyphemyl)-1*H*-benzo[*d*]imidazole. | 30 |
| Fig. S29: ^13^CNMR of the 2-(1*H*-benzo[*d*]imidazole-2-yl) phenol). | 31 |
| Fig. S30: ^13^CNMR of the 2-(1*H*-benzo[*d*]imidazole-2-yl) methoxyphenol). | 32 |

**1. Experimental Details**

**Materials and Methods**

All substrates, including ethanol, tetraethyl orthosilicate (TEOS), Pluronic P123, bis[3-(trimethoxysilyl) propyl] amine (BTPA), hydrochloric acid (HCl), lithium fluoride (LiF), MAXphase (Ti_3_AlC_2_), 3-aminopropyl triethoxysilane, and aldehydes, were purchased from Sigma, Aldrich, and Merck Chemical Companies. Additionally, pure distilled H_2_O and 96% EtOH were used as solvents.

**Characterization**

A UV lamp emitting light at a wavelength of 254 nm was utilized to conduct the thin-layer chromatography (TLC) experiment. The identification of the products was achieved through the use of a Shimadzu FTIR 8400S spectrometer, employing potassium bromide disks. Furthermore, ^1^HNMR spectra were recorded using a Bruker Avance 500 in DMSO-d6 solvent at ambient temperature. The melting points were measured using a 9100 Electrothermal apparatus and were presented without any corrections. The reported yields are calculated based on the products obtained after the purification process.

**2. Supplementary Figures**


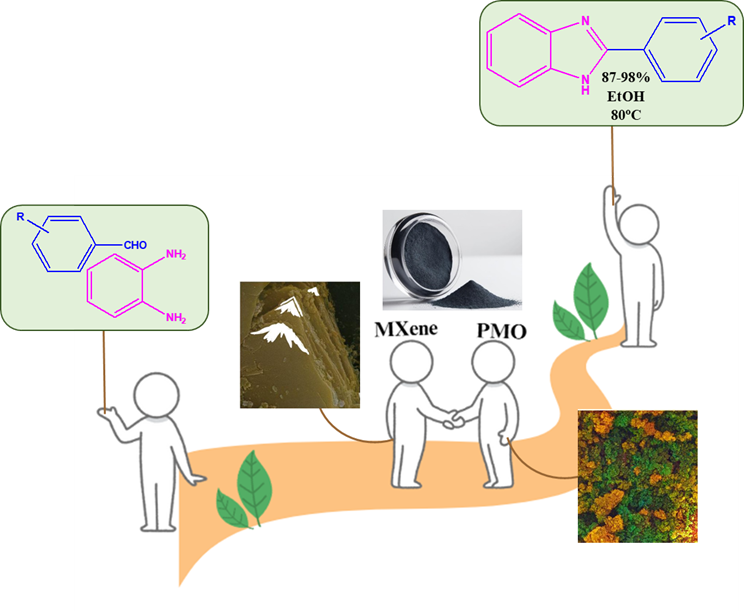


**Fig S1**: General scheme of the PMO@MXene nanocatalyst synthesis and its use in the synthesis of 2-(aryl)-1*H*-benzo[d]imidazole derivatives (Graphical abstract).

**Scheme S1**: Commercial drugs containing benzimidazole ring.


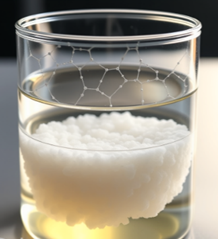


**Scheme S2**: PMO framework diagram.

**
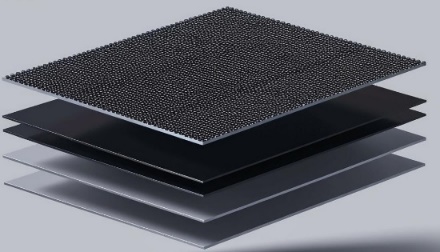
**

**Scheme S3**: Structure of MXene.


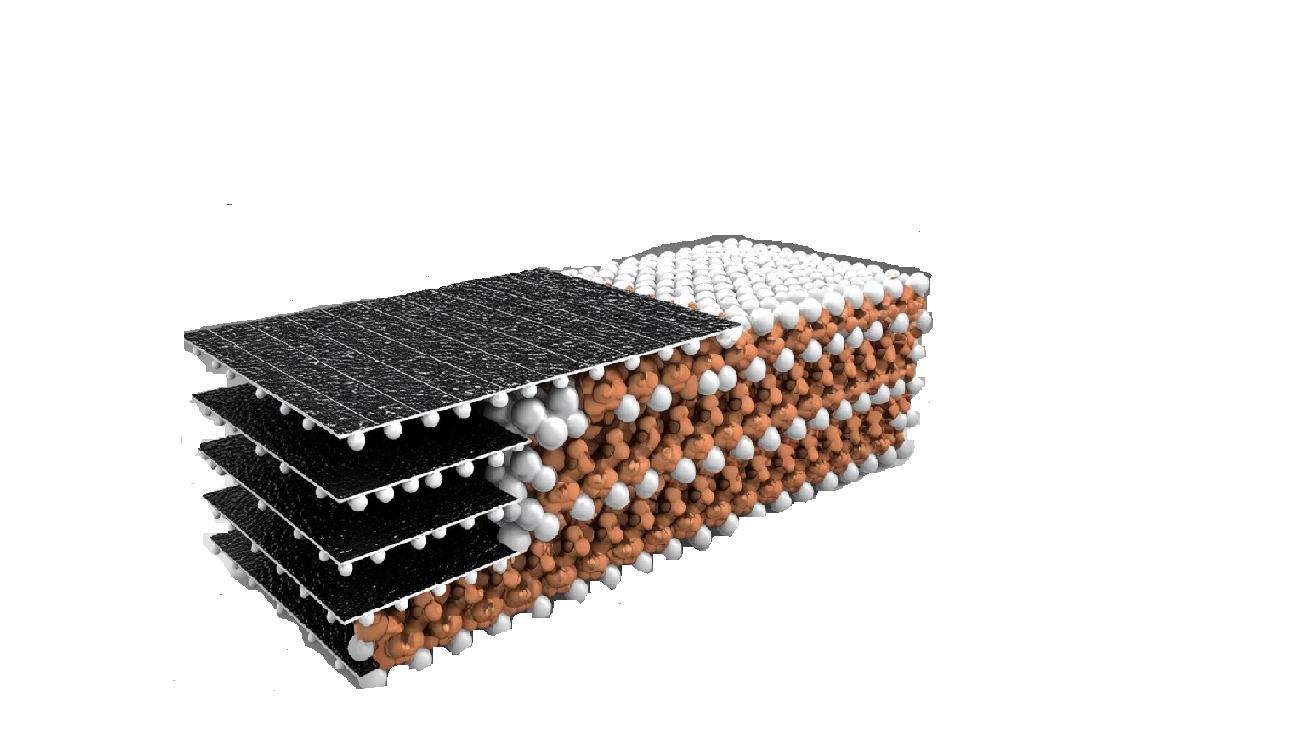


**Scheme S4**: The structure of the PMO@MXene nanocomposite.


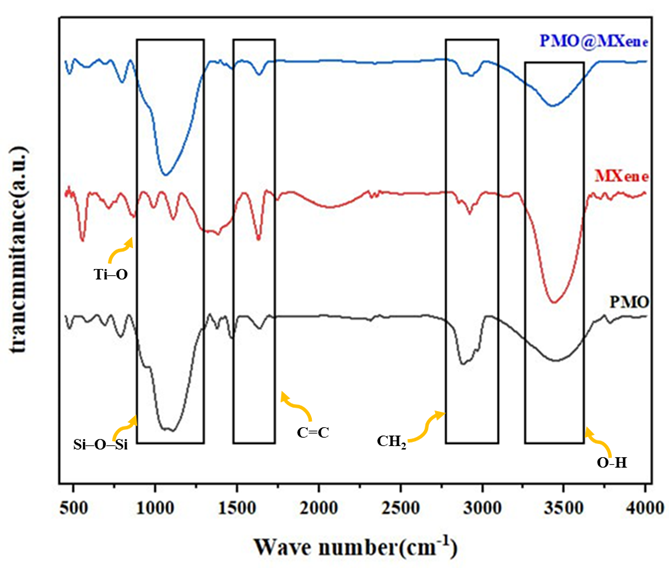


**Fig S2**: FT-IR of PMO, MXene, and the PMO@MXene nanocomposite.


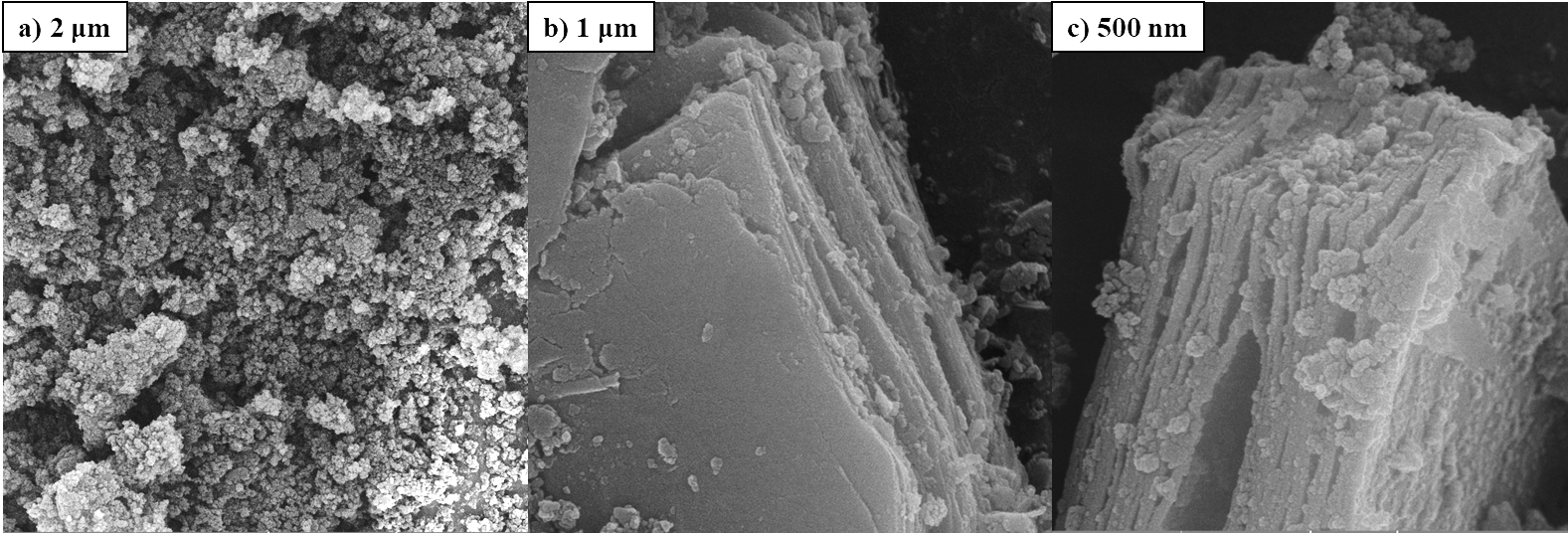


**Fig S3**: FESEM images of **a)** PMO (2 μm), **b)** MXene (1 μm), and **c)** the PMO@MXene nanocomposite (500 nm).


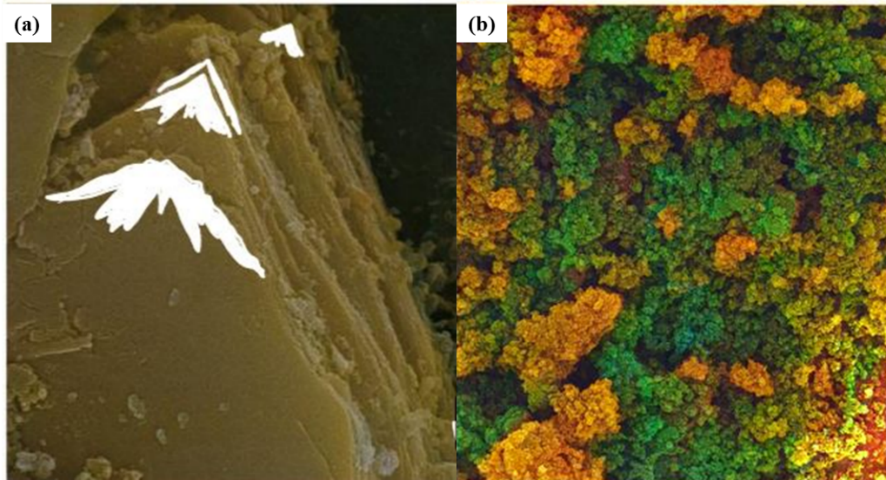


**Fig S4:** FESEM image of (a) MXene, like a snowy mountain and (b) PMO like a jungle designed by the Nanoart method.

**
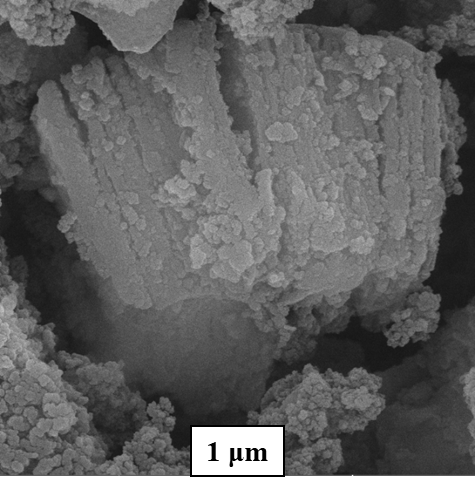
**

**Fig. S5**: FESEM image of the recycled PMO@MXene nanocomposite (1 μm).


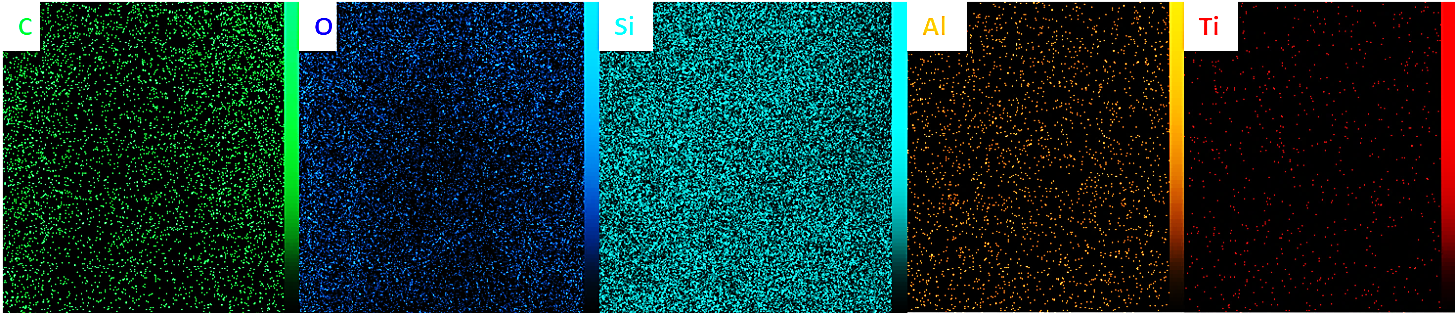


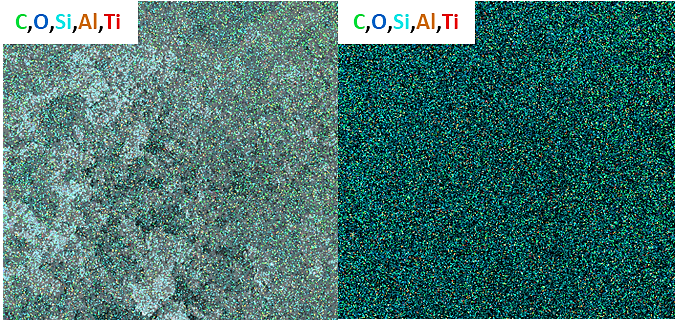


**Fig S6**: EDS elemental mapping analysis of the PMO@MXene nanocomposite.

**Intensity (a.u.)**

**b**


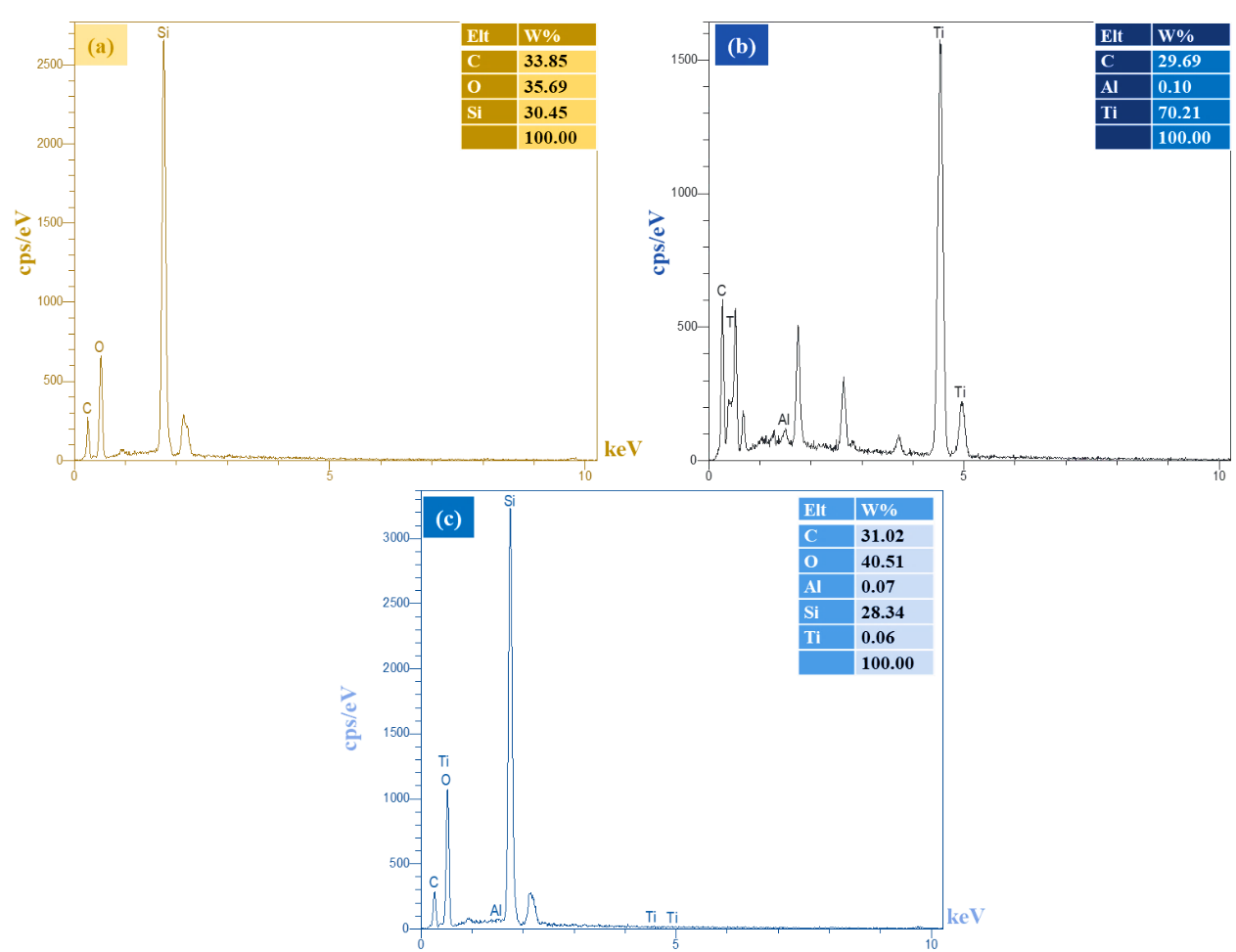


**Fig S7**: Elemental analysis by energy-dispersive X-ray spectroscopy (EDS) of **a)** PMO, **b)** MXene, and **c)** the PMO@MXene nanocomposite**.**


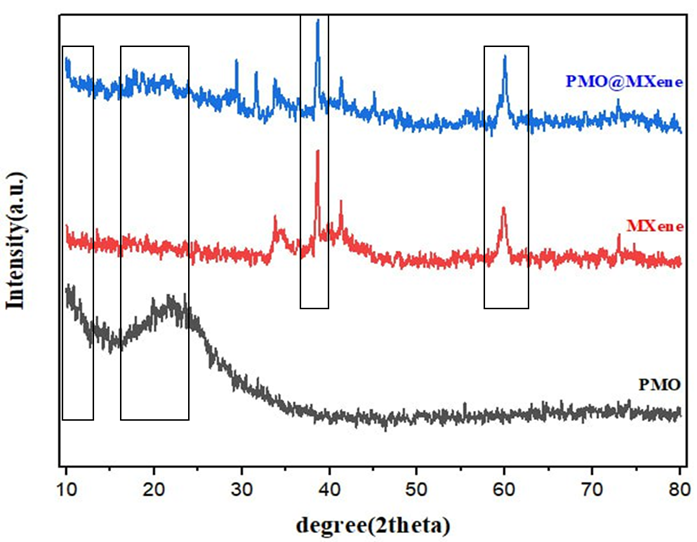


**Fig. S8:** X-ray diffraction patterns of PMO**,** MXene, and the PMO@MXene nanocomposite.


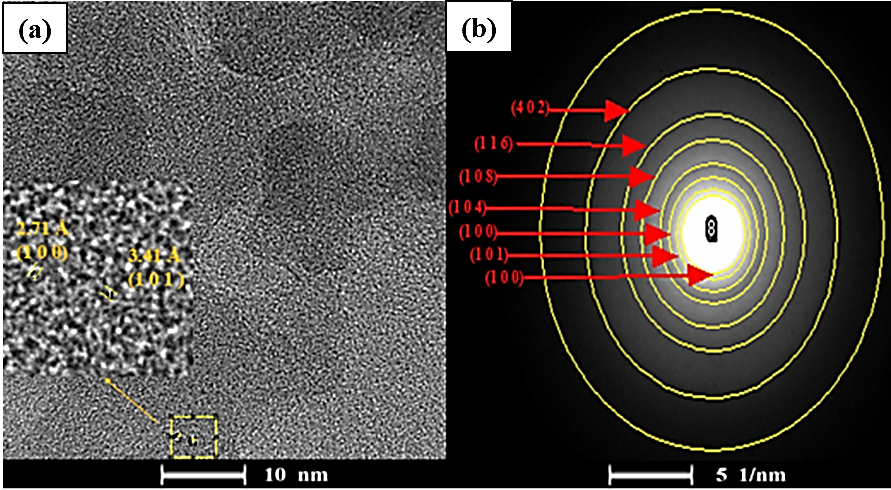


**Fig. S9:** HRTEM analysis (a), and SAED analysis (b) of the PMO@MXene nanocomposite.


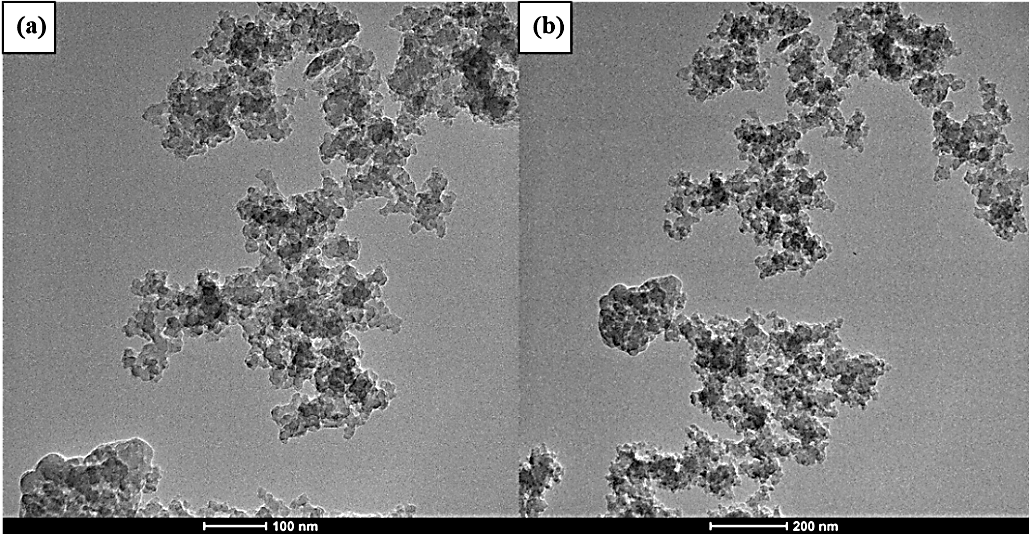


**Fig. S10:** The TEM images of the (a) PMO@MXene and (b) recycled PMO@MXene nanocomposite.

| **Table S1**: Exploring optimized conditions for the one-pot synthesis of 2-(aryl)-1-*H*-benzo[d]imidazole derivatives obtained from the reaction of 2-chlorobenzaldehyde (**3a**) and 1,2-phenylene diamine (**2**). | | | | | |
| --- | --- | --- | --- | --- | --- |
| **Entry** | **Catalyst loading**  **(mg)** | **Solvent**  **(mL)** | **Temperature (^o^C)** | **Time**  **(min.)** | **Yield^a,b^**  **(%)** |
| 1 | - | H_2_O (2.0) | r.t | 45 | - |
| 2 | PMO (2.0) | H_2_O (2.0) | r.t | 45 | 15 |
| 3 | MXene (2.0) | H_2_O (2.0) | r.t | 60 | 23 |
| 4 | PMO@MXene (2.0) | H_2_O (2.0) | r.t | 40 | 25 |
| 5 | PMO@MXene (2.0) | MeOH (2.0) | r.t | 45 | 28 |
| 6 | PMO@MXene (2.0) | EtOH (2.0) | r.t | 45 | 38 |
| 7 | PMO@MXene (2.0) | EtOAc (2.0) | r.t | 45 | 26 |
| 8 | PMO@MXene (2.0) | CH_2_Cl_2_ (2.0) | r.t | 45 | 32 |
| 9 | PMO@MXene (2.0) | H_2_O/EtOH (2.0) | r.t | 45 | 30 |
| 10 | PMO@MXene (2.0) | - | r.t | 45 | 57 |
| 11 | PMO@MXene (2.0) | - | 80 | 30 | 68 |
| **12** | **PMO@MXene (5.0)** | **EtOH (2.0)** | **80** | **10** | **98** |
| 13 | PMO@MXene(7.5) | - | 80 | 15 | 92 |
| 14 | PMO@MXene (5.0) | - | 80 | 15 | 31 |
| 15 | - | - | 80 | 15 | - |
| ****  ^a^Reaction conditions: aldehyde (**1a**, 1.0 mmol), 1,2-phenylene diamine (**2**, 1.0 mmol), and 2.0 mL EtOH as a solvent. ^b^The yield refers to the isolated pure product **3a**. | | | | | |

| **Table S2:** Synthesis of diverse 2-(aryl)-1*H*-benzimidazole (3a-l) using various aldehydes in the presence of the PMO@MXene nanocomposite.^a^ | | | | | | | | |
| --- | --- | --- | --- | --- | --- | --- | --- | --- |
| 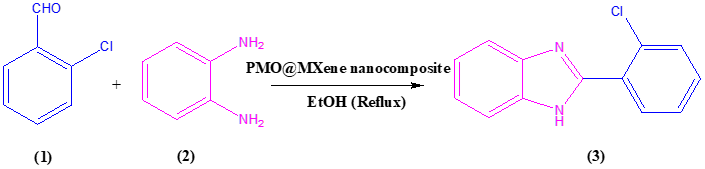 | | | | | | | | |
| **Entry** | **Aldehyde** | **Product** | **Time (min.)** | **Yield^b^ (%)** | **M.P.**  **(^o^C)** | **M.P.**  **(^o^C) (Ref.)** | **TON** | **TOF** |
| 1 |   1a |   3a | 10 | 98 | 231-234 | 232-234 [63] | 4600938.9 | 460093.8 |
| 2 |   1b |   3b | 20 | 90 | 205-206 | 203-204 [64] | 4225325.1 | 211267.6 |
| 3 |   1c |   3c | 20 | 90 | 265-270 | 264-266 [65] | 4225325.1 | 211267.6 |
| 4 |   1d |   3d | 15 | 95 | 292-293 | 291-293 [63] | 4460093.8 | 297339.5 |
| 5 |   1e | 3e | 20 | 89 | 310-312 | 312-314 [64] | 4178403.7 | 208920.1 |
| 6 |   1f |   3f | 10 | 96 | 283-285 | 283-284  [65] | 4507042.2 | 450704.2 |
| 7 |   1g |   3g | 15 | 87 | 286-292 | 290-292  [63] | 4084507.04 | 272300.4 |
| 8 |   1h |   3h | 10 | 92 | 223-226 | 225-226  [63] | 4319248.8 | 431924.8 |
| 9 |   1i |   3i | 15 | 92 | 179-185 | New sample  [-] | 4319248.8 | 287949.9 |
| 10 |   1j |   3j | 10 | 87 | 295-298 | New sample  [-] | 4084507.04 | 408450.7 |
| 11 |   1k |   3k | 10 | 91 | 267-270 | 268-270  [63] | 4272300.4 | 427230.04 |
| 12 |   1l |   3l | 20 | 89 | 190-230 | New sample  [-] | 4178403.7 | 208920.1 |
| ^a^Reaction conditions: aromatic aldehyde (1a–l, 1.0 mmol), 1,2-phenylene diamine (2, 1.0 mmol), PMO@MXene (5.0 mg), and EtOH (2.0 mL) under reflux (80 ^o^C) conditions. ^b^The yields refer to the isolated pure products. | | | | | | | | |


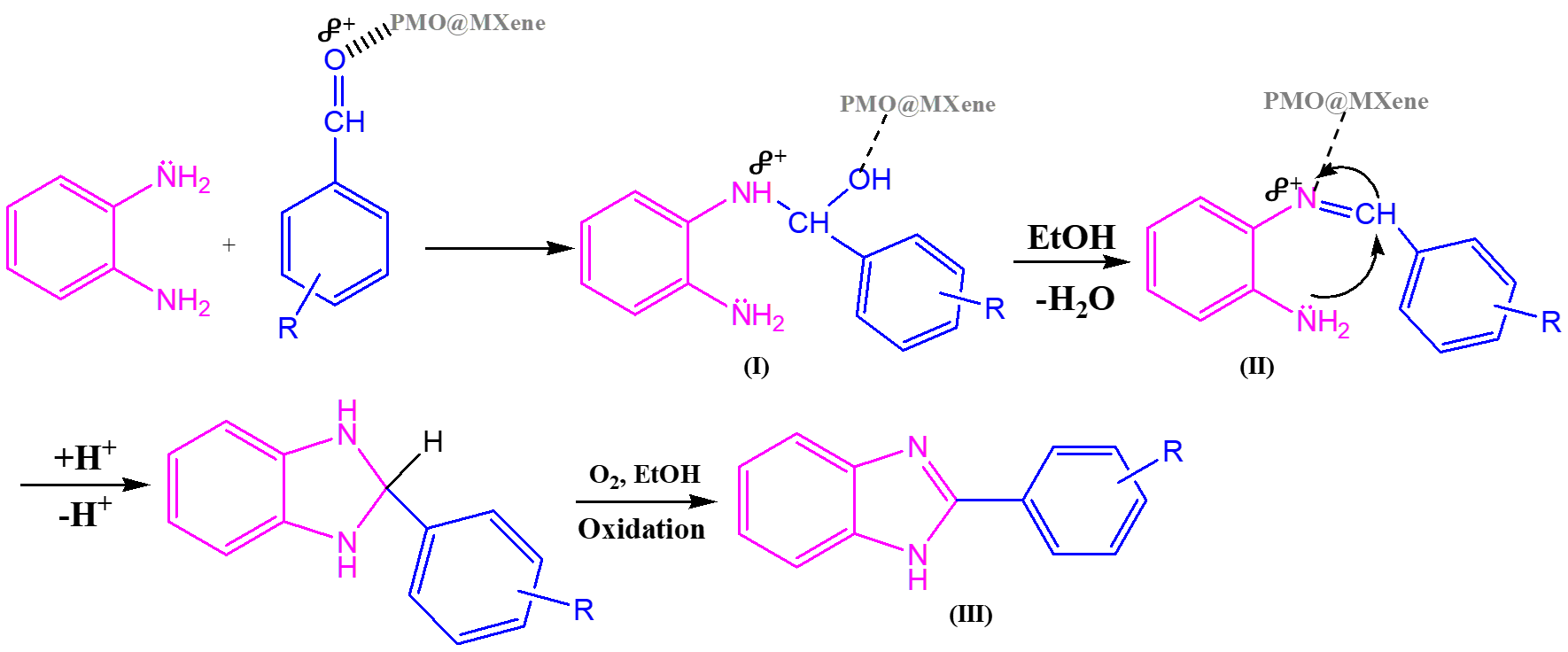


**Fig S11**: Proposed mechanism for the synthesis of 2-(aryl)-1*H*-benzo[*d*]imidazole derivatives in the presence of the PMO@MXene nanocatalyst.


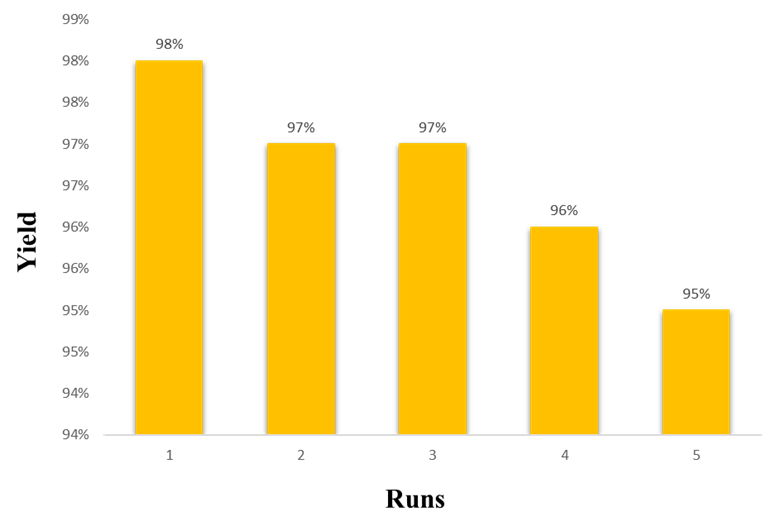


**Fig S12**: The results of recyclability and reusability of the PMO@MXene nanocomposite in the model reaction.


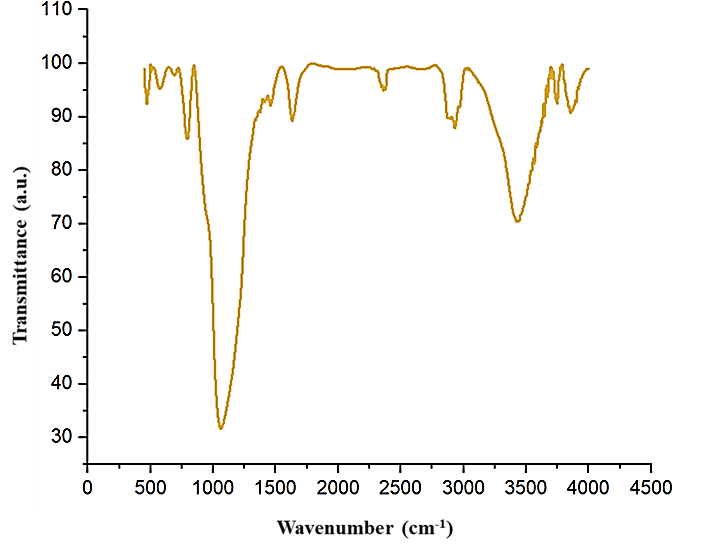


**Fig. S13**: FT-IR spectrum of the recycled PMO@MXene nanocomposite after 5 runs.


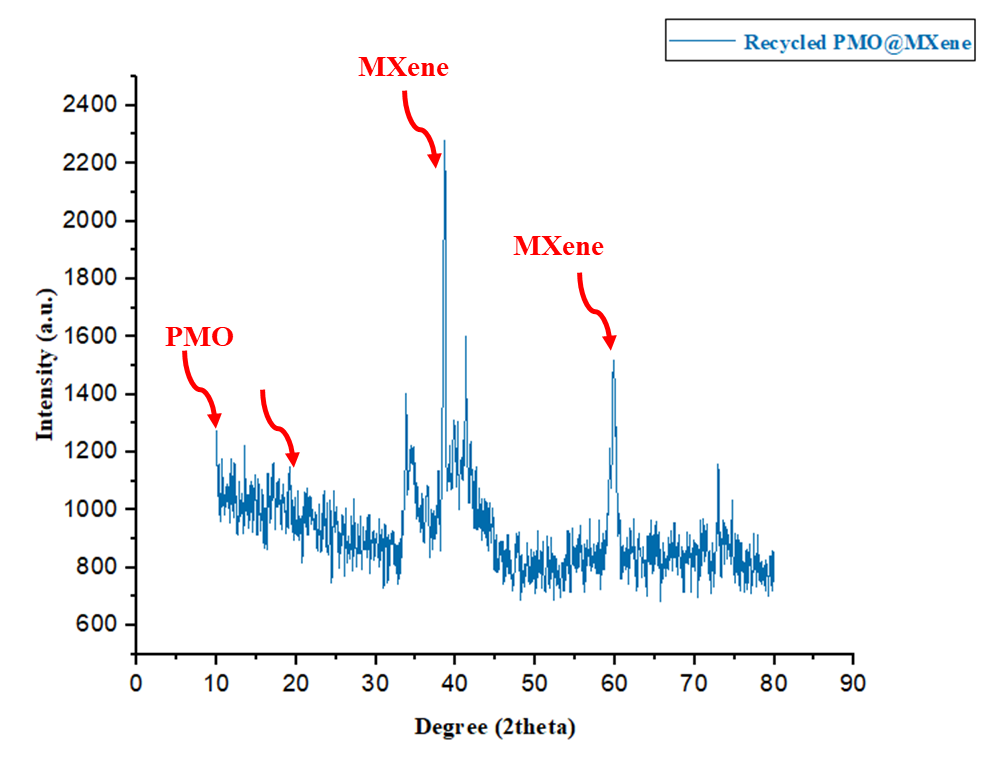


**Fig. S14:** X-ray diffraction patterns of the recycled PMO@MXene nanocomposite.

| **Table S3:** Comparison of the effectiveness of PMO@MXene in the promotion of the synthesis of 2-(aryl)-1*H*-benzimidazoles with some of the recently reported protocols.  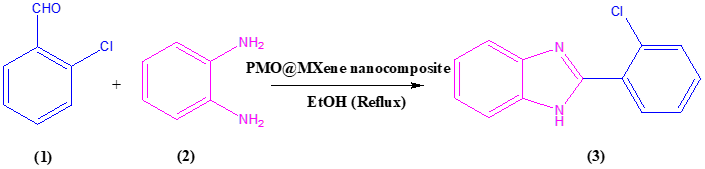 | | | | | |
| --- | --- | --- | --- | --- | --- |
| **Entry** | **Catalyst loading** | **Reaction conditions** | **Time (min.)** | **Yield (%)** | **Ref.** |
| **1** | In(OTF)_3_/ 5.0 mol% | r.t/Solvent-free | 30 | 95 | [68] |
| **2** | Na_3_AlF_6_/ 2.0 mol% | 50 ^o^C/ EtOH | 120 | 80 | [69] |
| **3** | LaCl_3_/ 10.0 mol% | r.t/ CH_3_CN | 120 | 95 | [70] |
| **4** | Dioxane dibromide 100.0 mol% | r.t/ CH_3_CN | 30-60 | 87 | [71] |
| **5** | Zn (OTf)_2_ 10.0 mol% | Reflux/ EtOH | 480 | 95 | [72] |
| **6** | PMO@MXene 5.0 mg | 80 ^o^C/ EtOH | 10 | 98 | This work |

The nitrogen adsorption–desorption isotherm of the PMO@MXene catalyst exhibited a type IV profile according to the IUPAC classification, which is characteristic of mesoporous materials. The BET surface area was calculated to be 92.9 m²/g, a value consistent with the single-point surface area (94.2 m²/g). Moreover, the Langmuir surface area was determined to be 136.2 m²/g. The t-plot analysis further revealed a micropore surface area of 62.0 m²/g and an external surface area of 30.9 m²/g. These findings confirm the coexistence of both microporous and mesoporous structures in PMO@MXene, where the layered structure of MXene contributes significantly to the external surface area.


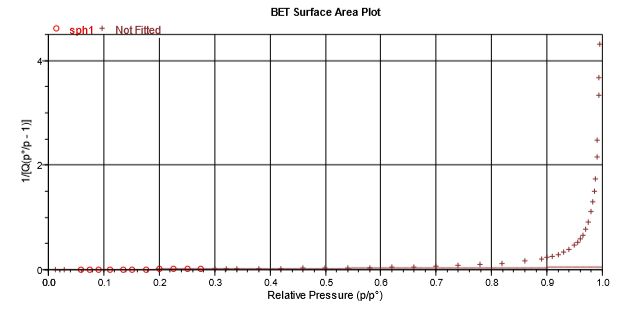


***p/p_0_***

**BET-Plot**

***p/V_0_(p-p_0_)***

**Fig. S15:** Brunaur-Emmet-Teller (BET) surface area analysis of the PMO@MXene nanocomposite.


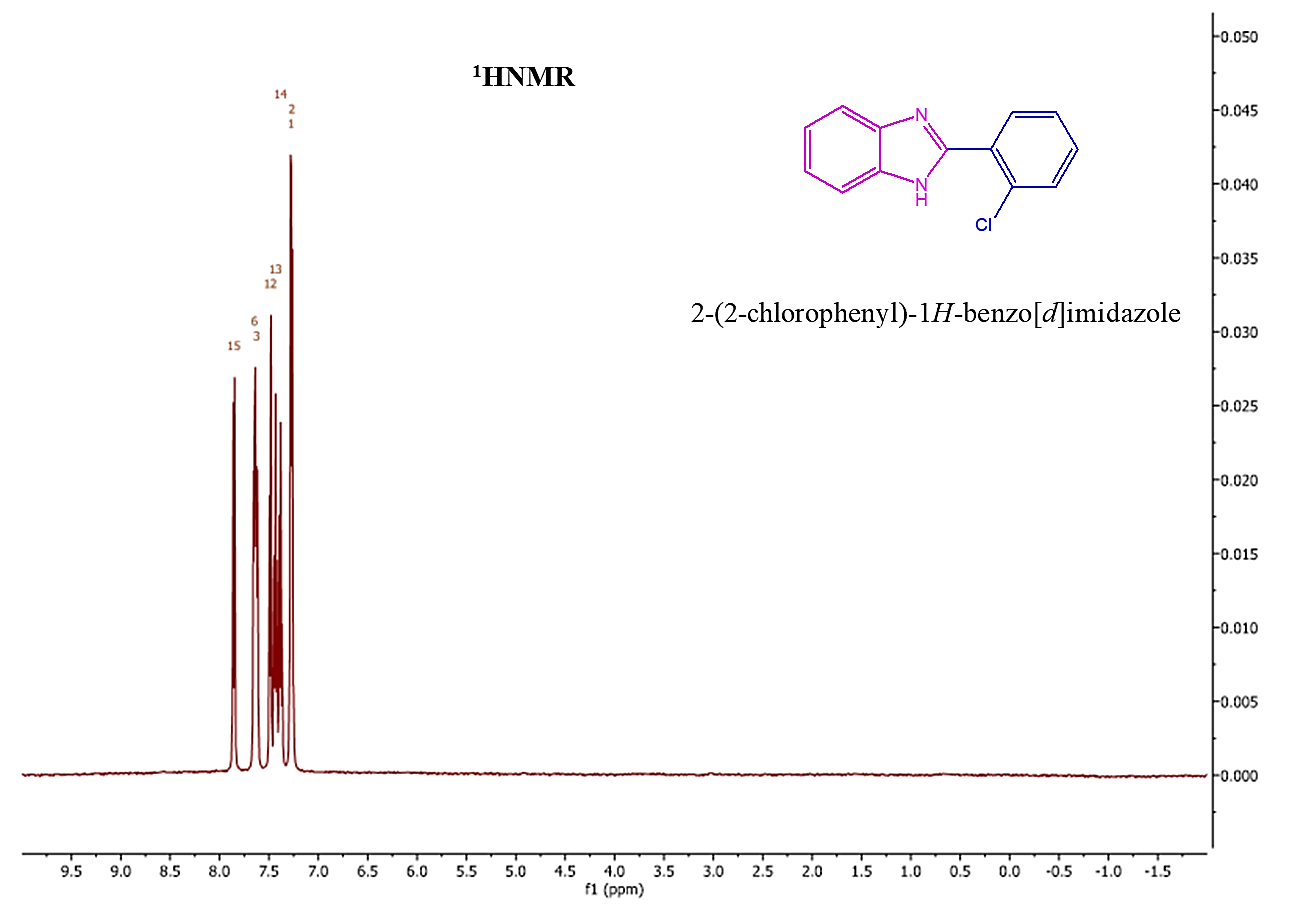


**Fig. S16:** ^1^HNMR of the 2-(2-chlorophenyl)-1*H*-benzo[*d*]imidazole.


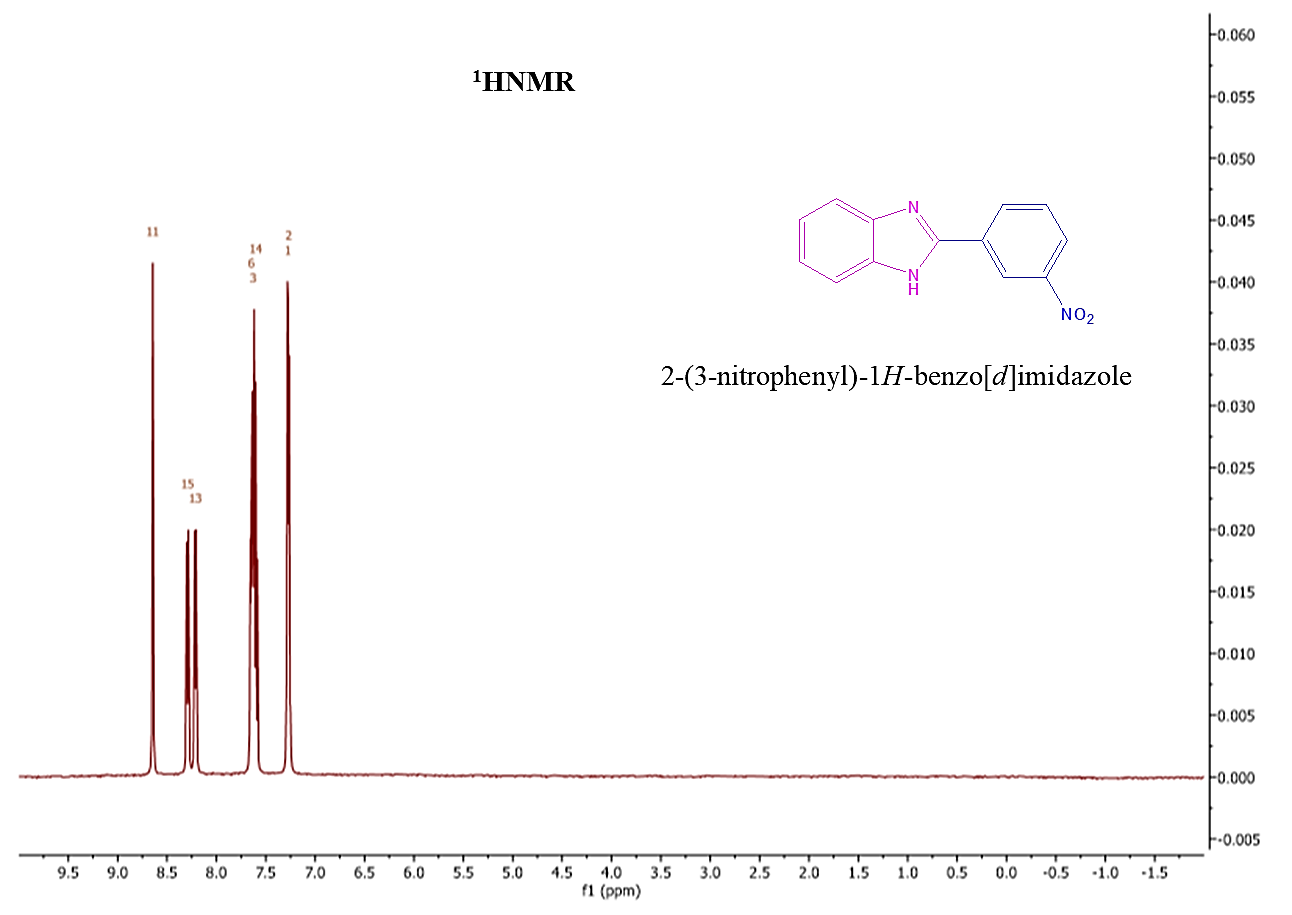


**Fig. S17:** ^1^HNMR of the 2-(3-nitrophenyl)-1*H*-benzo[*d*]imidazole.


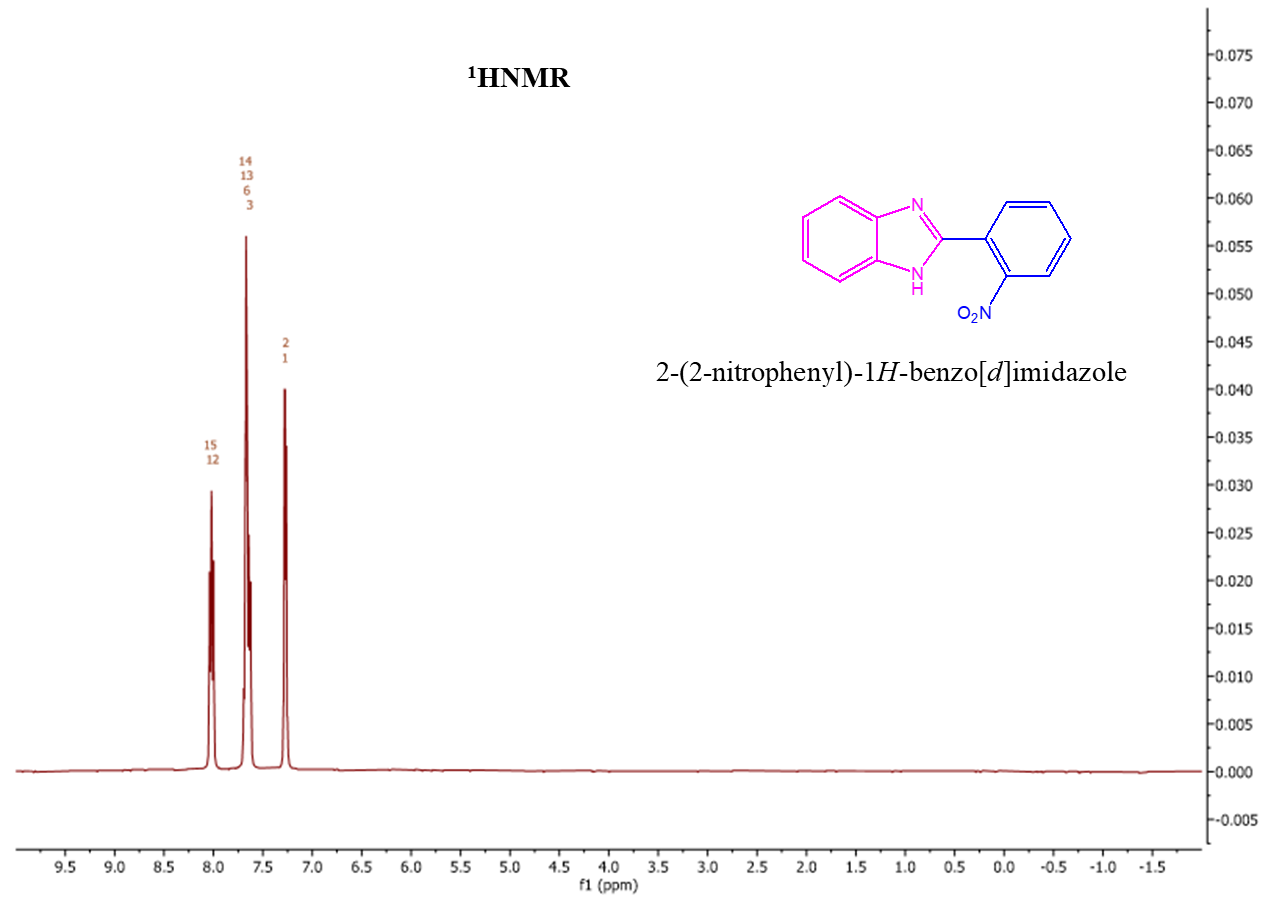


**Fig. S18:** ^1^HNMR of the 2-(2-nitrophenyl)-1*H*-benzo[*d*]imidazole.


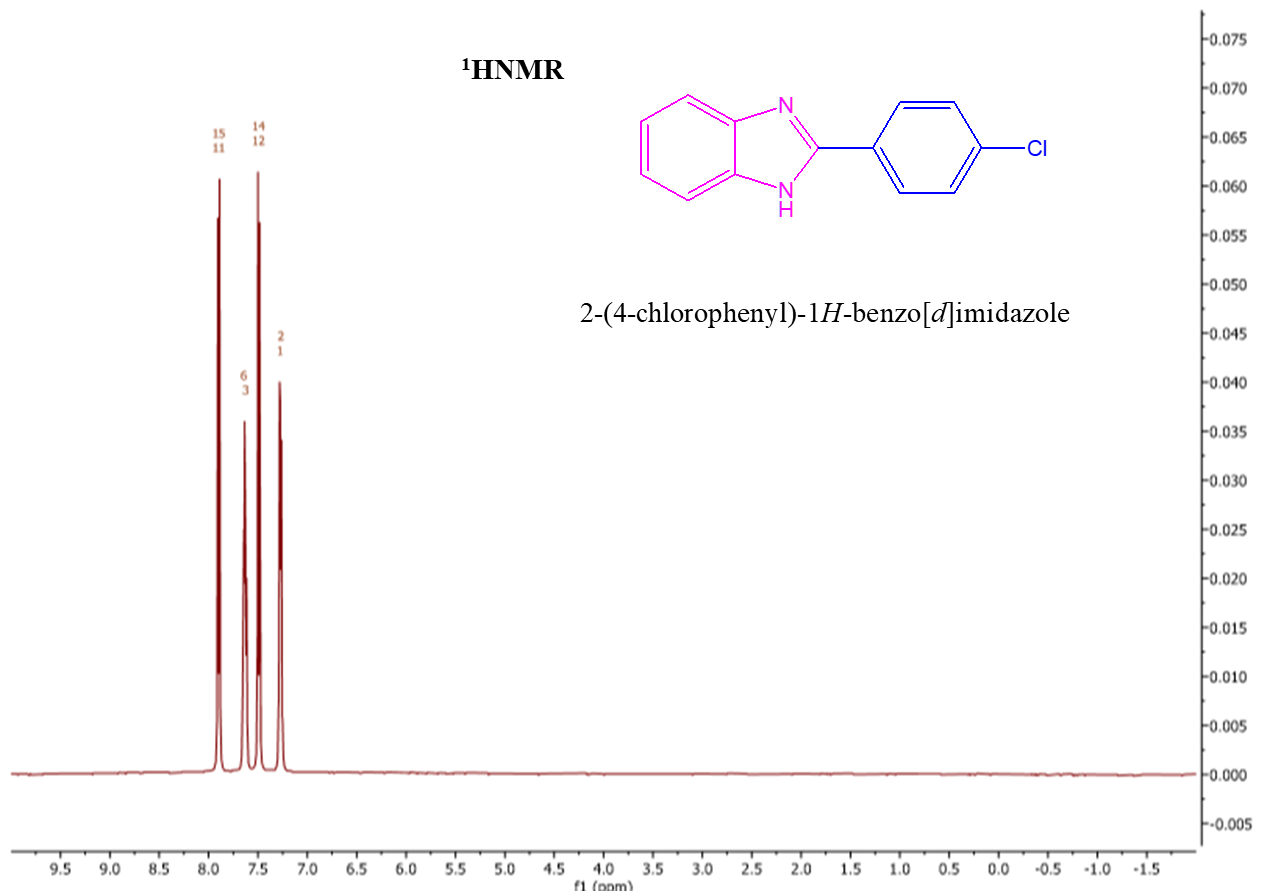


**Fig. S19:** ^1^HNMR of the 2-(4-chlorophenyl)-1*H*-benzo[*d*]imidazole.


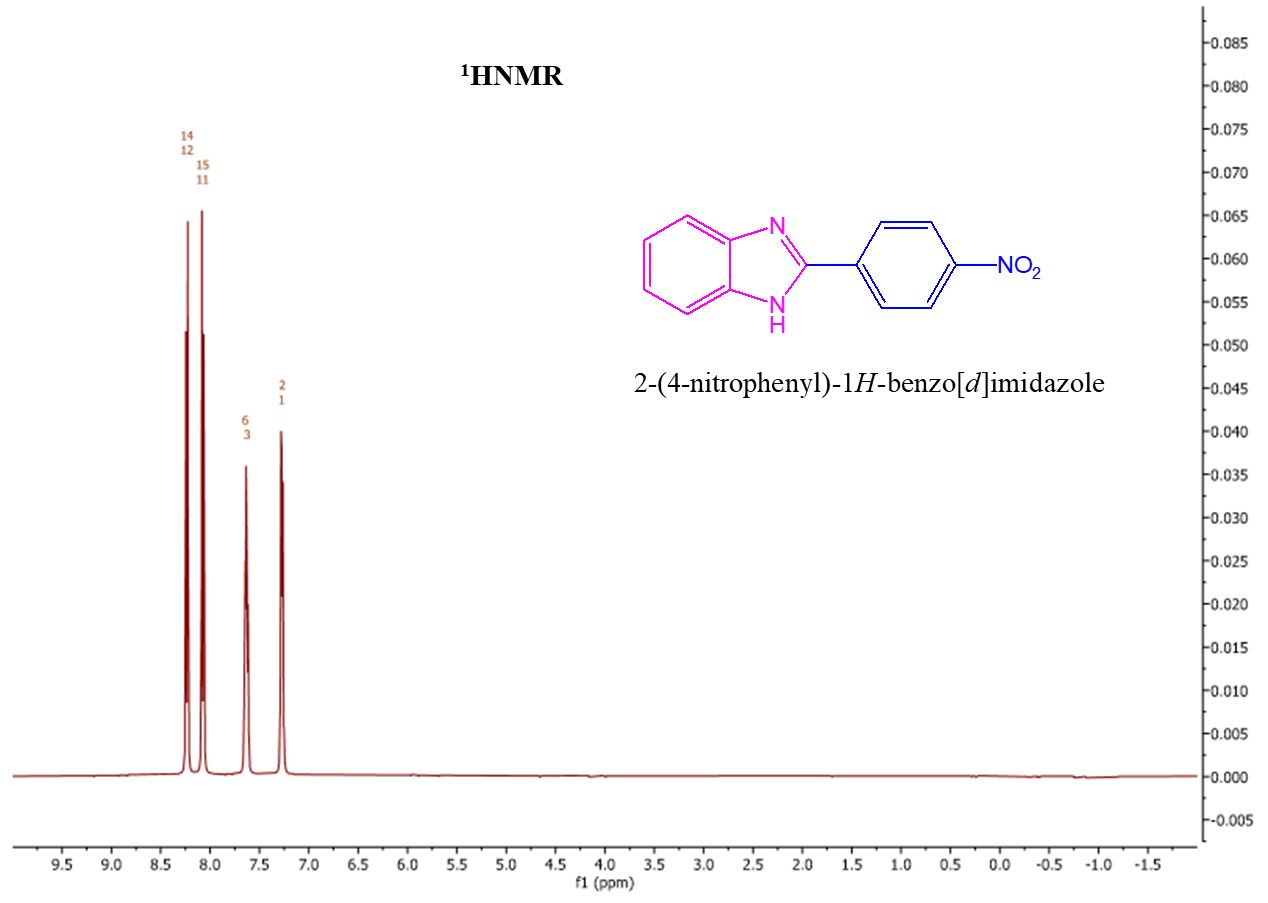


**Fig. S20:** ^1^HNMR of the 2-(4-nitrophenyl)-1*H*-benzo[*d*]imidazole.


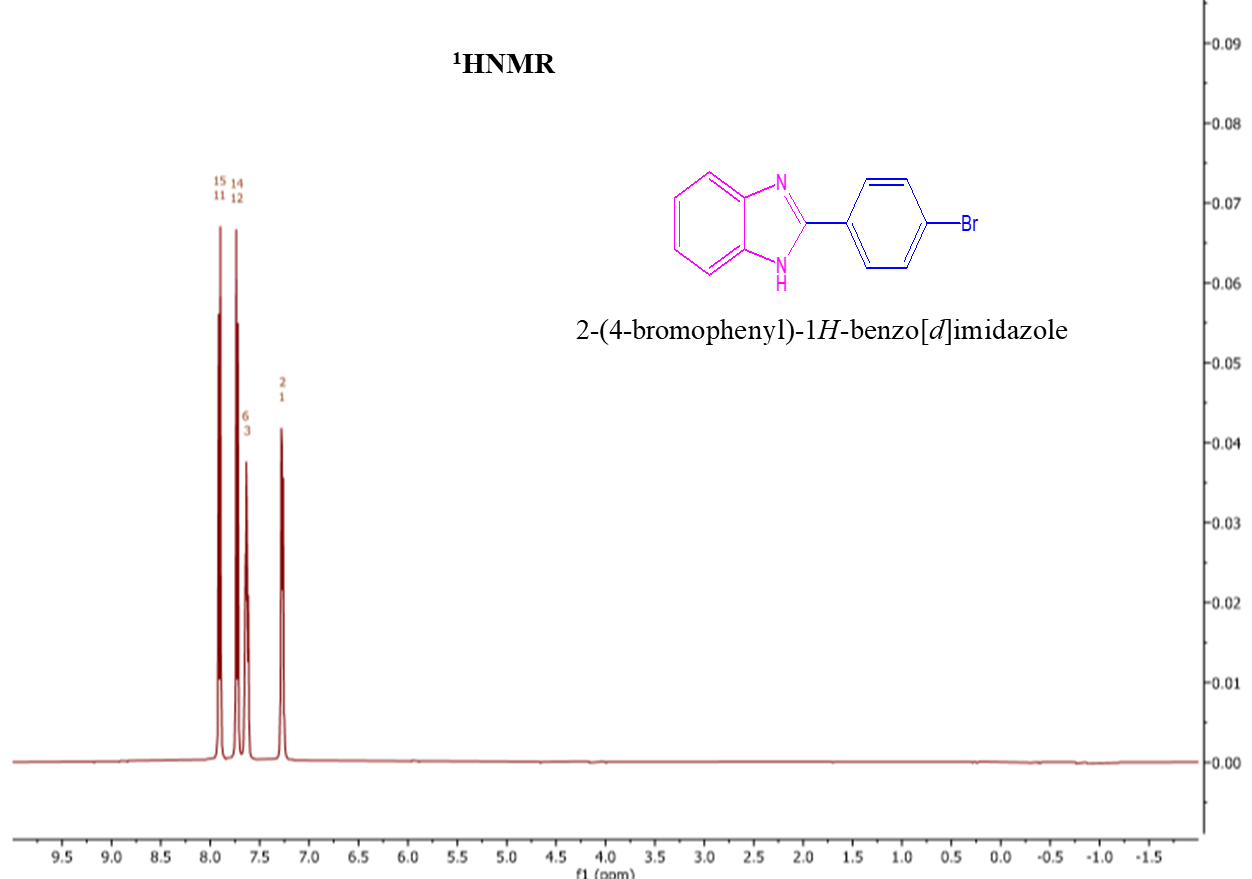


**Fig. S21:** ^1^HNMR of the 2-(4-bromophenyl)-1*H*-benzo[*d*]imidazole.


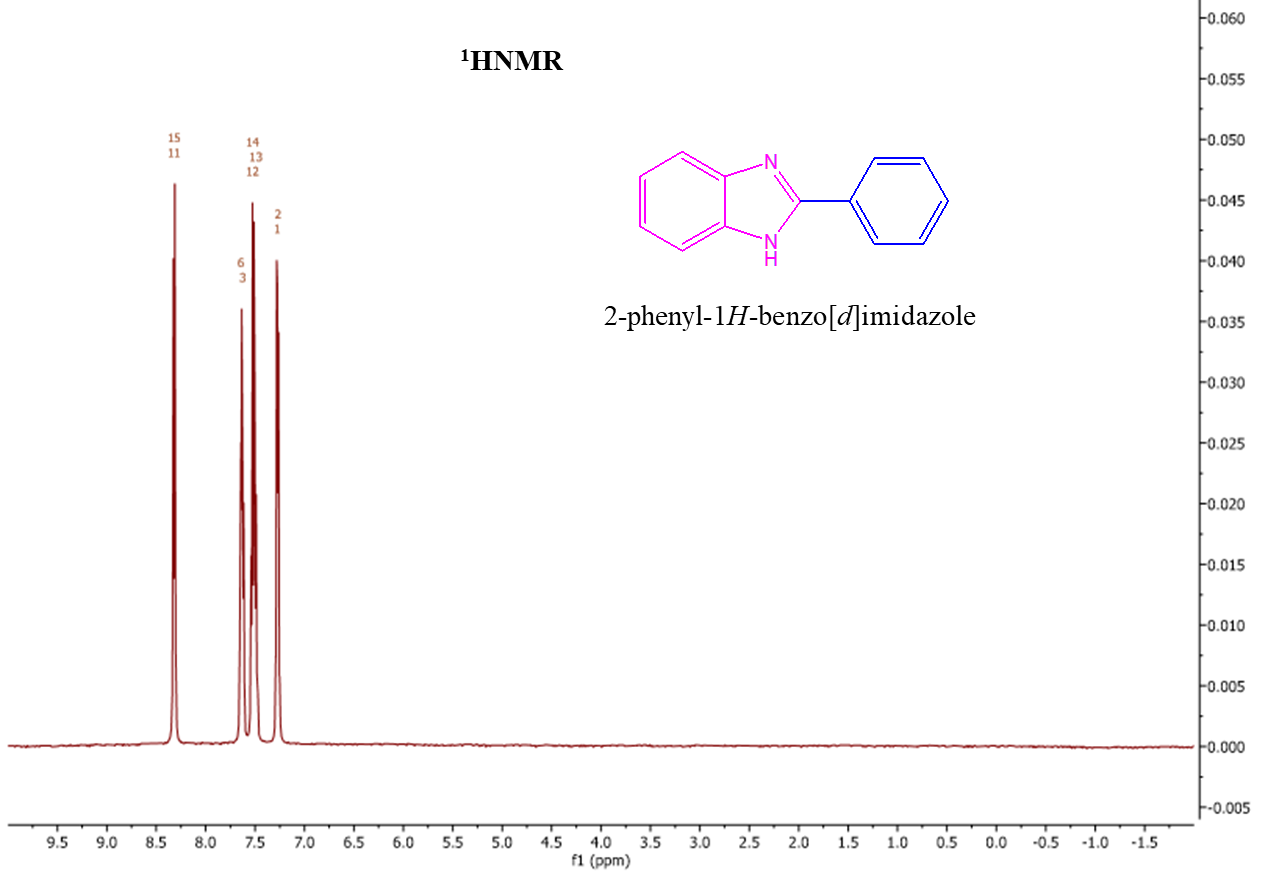


**Fig. S22**: 1HNMR of the 2-phenyl-1*H*-benzo[*d*]imidazole.


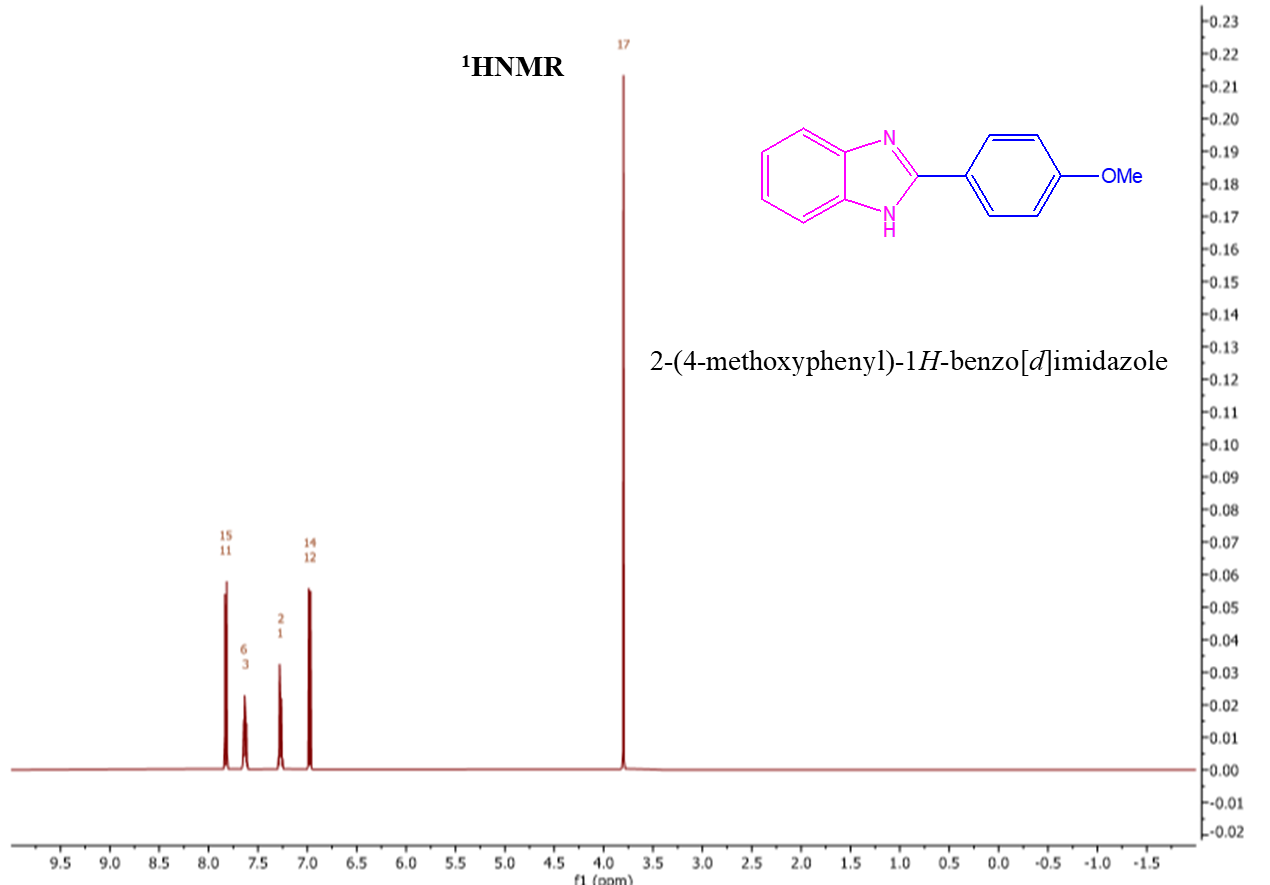


**Fig. S23:** ^1^HNMR of the 2-(4-methoxyphenyl)-1*H*-benzo[*d*]imidazole.


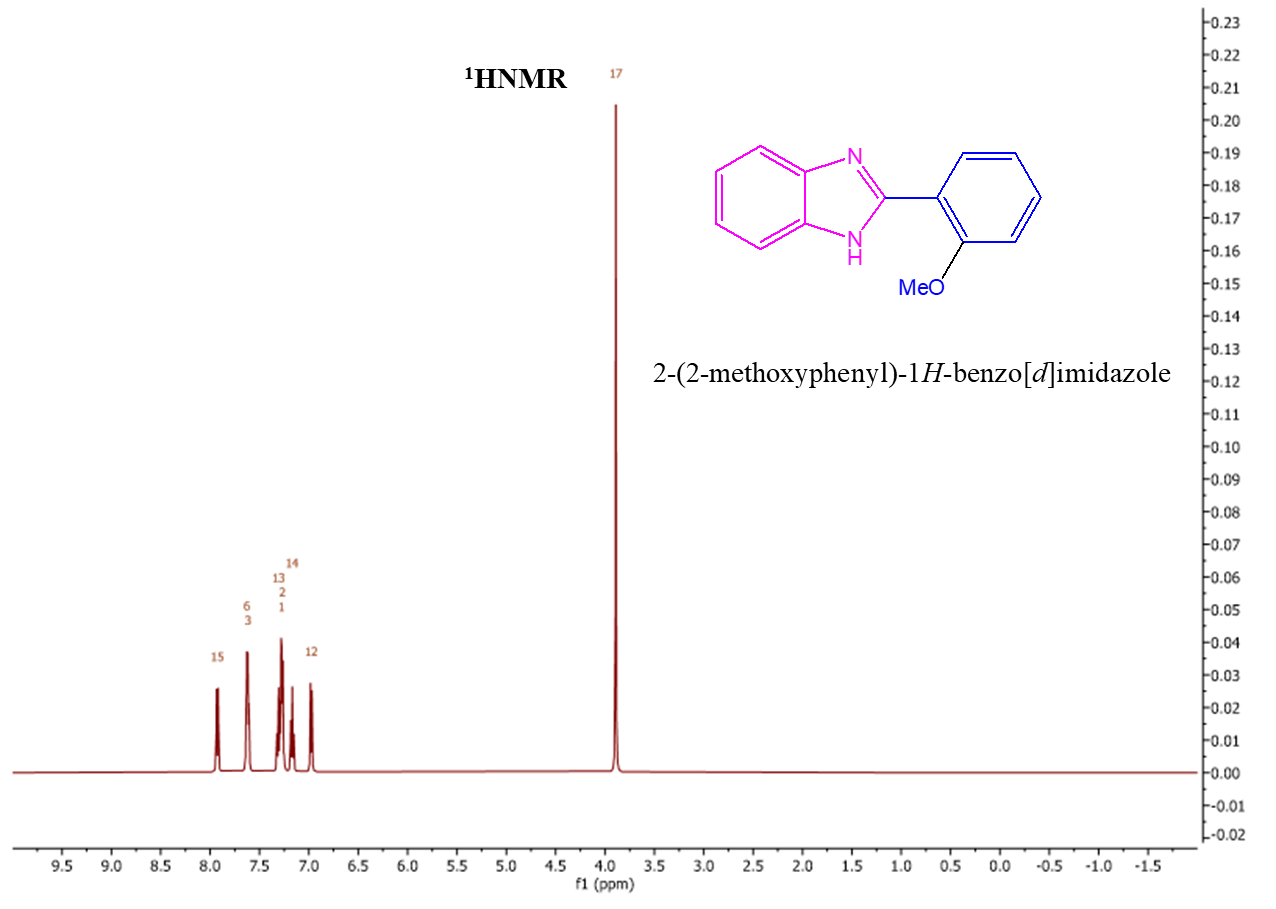


**Fig. S24:** ^1^HNMR of the 2-(2-methoxyphenyl)-1*H*-benzo[*d*]imidazole.


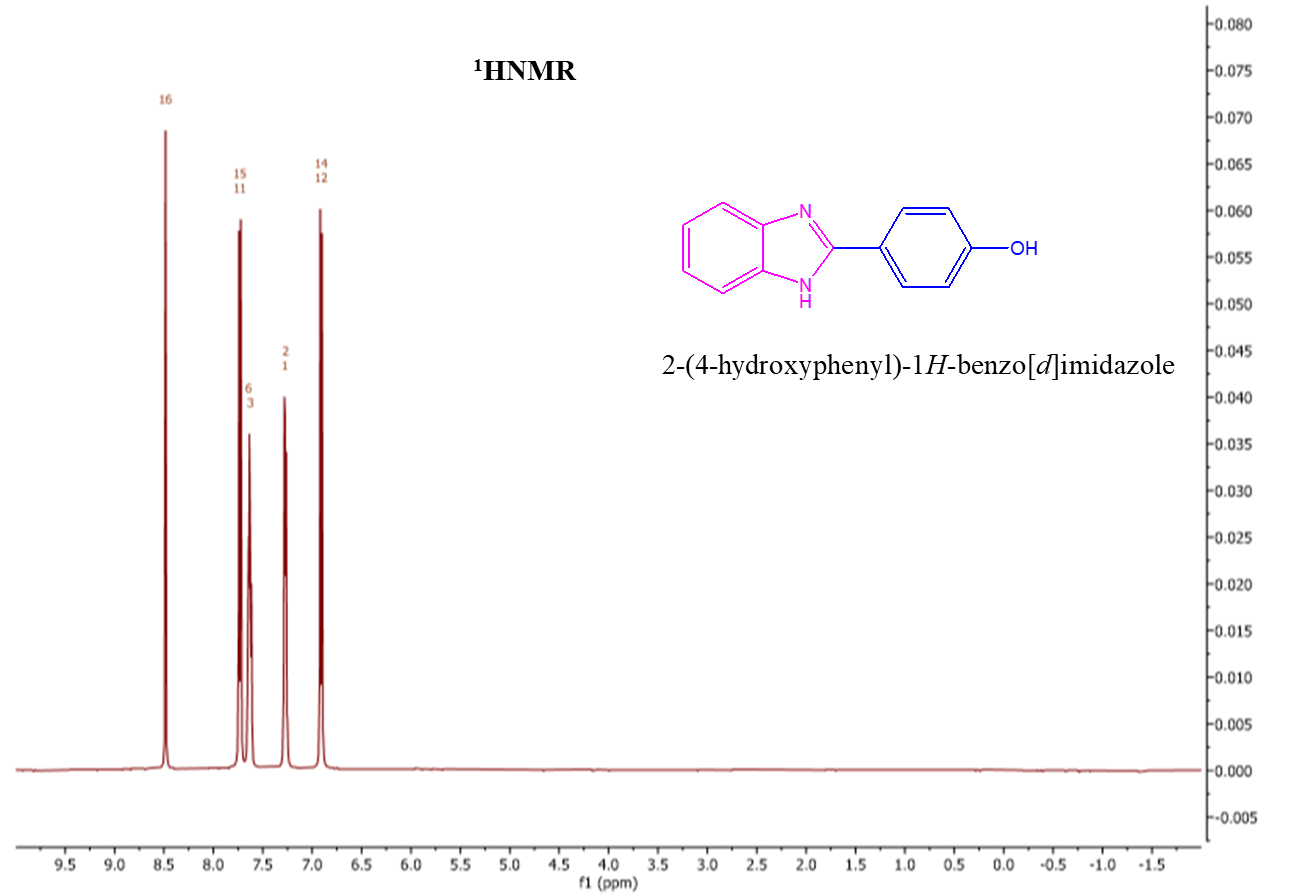


**Fig. S25:** ^1^HNMR of the 2-(4-hydroxyphenyl)-1*H*-benzo[*d*]imidazole.


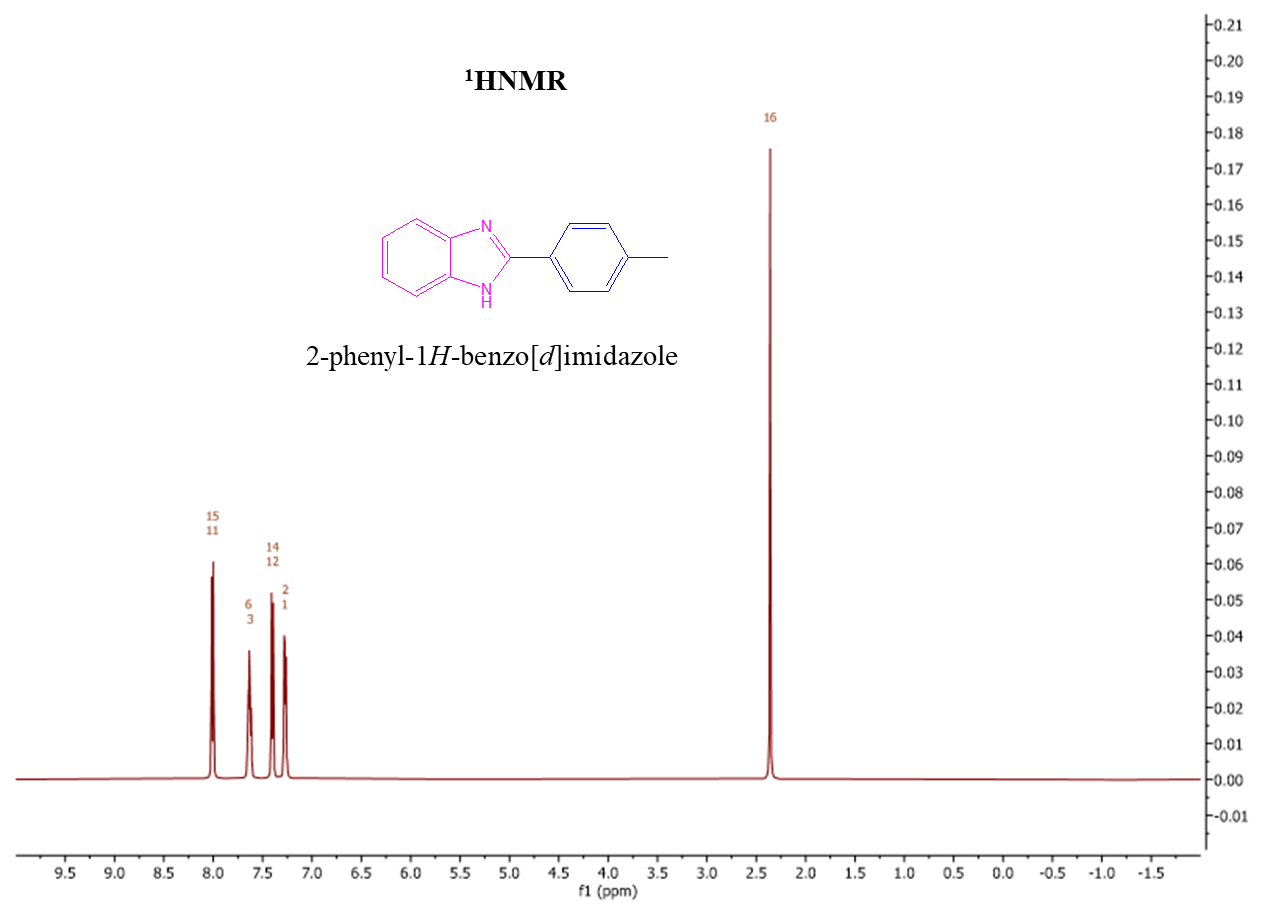


**Fig. S26**: ^1^HNMR of the 2-phenyl-1*H*-benzo[*d*]imidazole


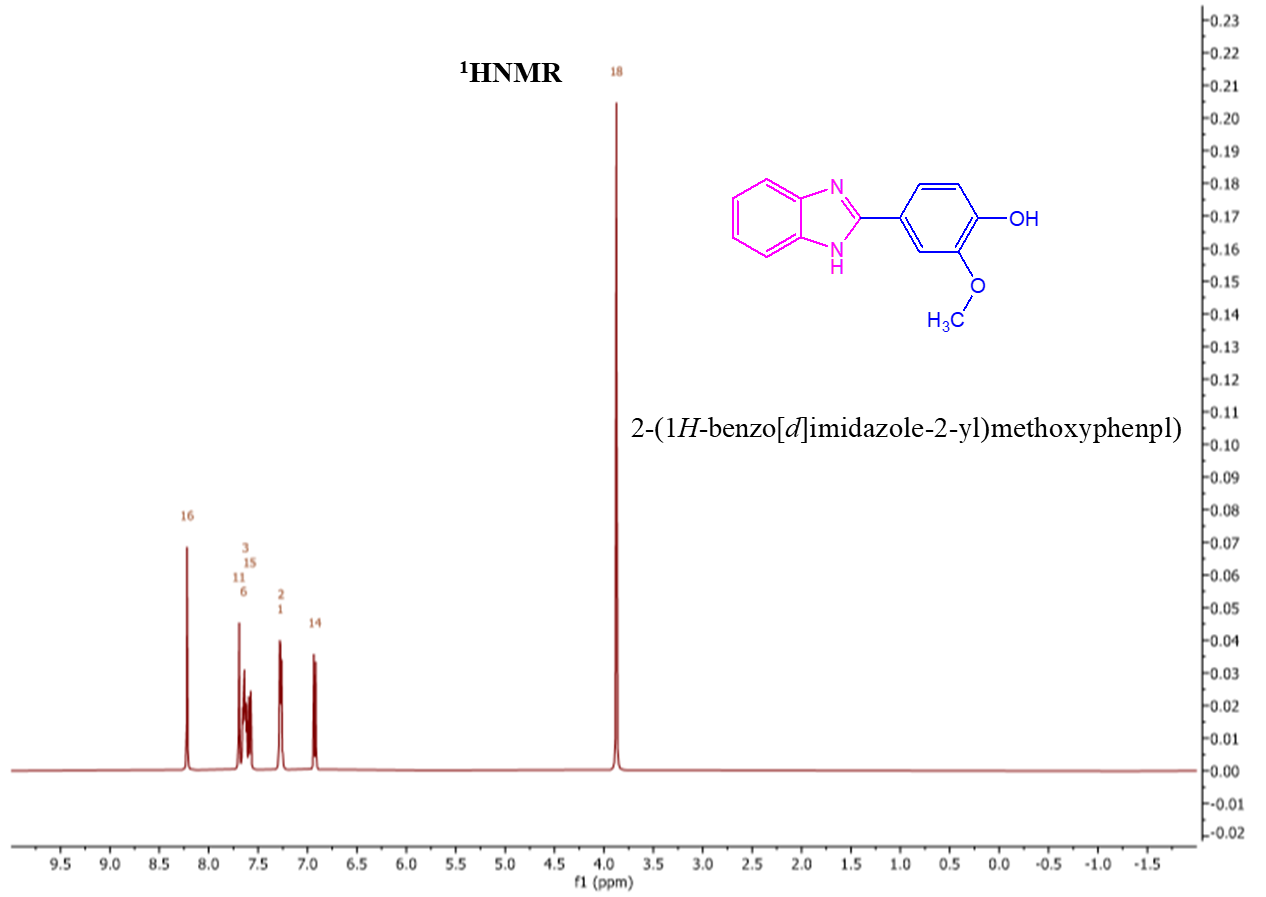


**Fig. S27**: ^1^HNMR of the 2-(1*H*-benzo[*d*]imidazole-2-yl)methoxyphenpl)

**^13^CNMR data of the new samples:**


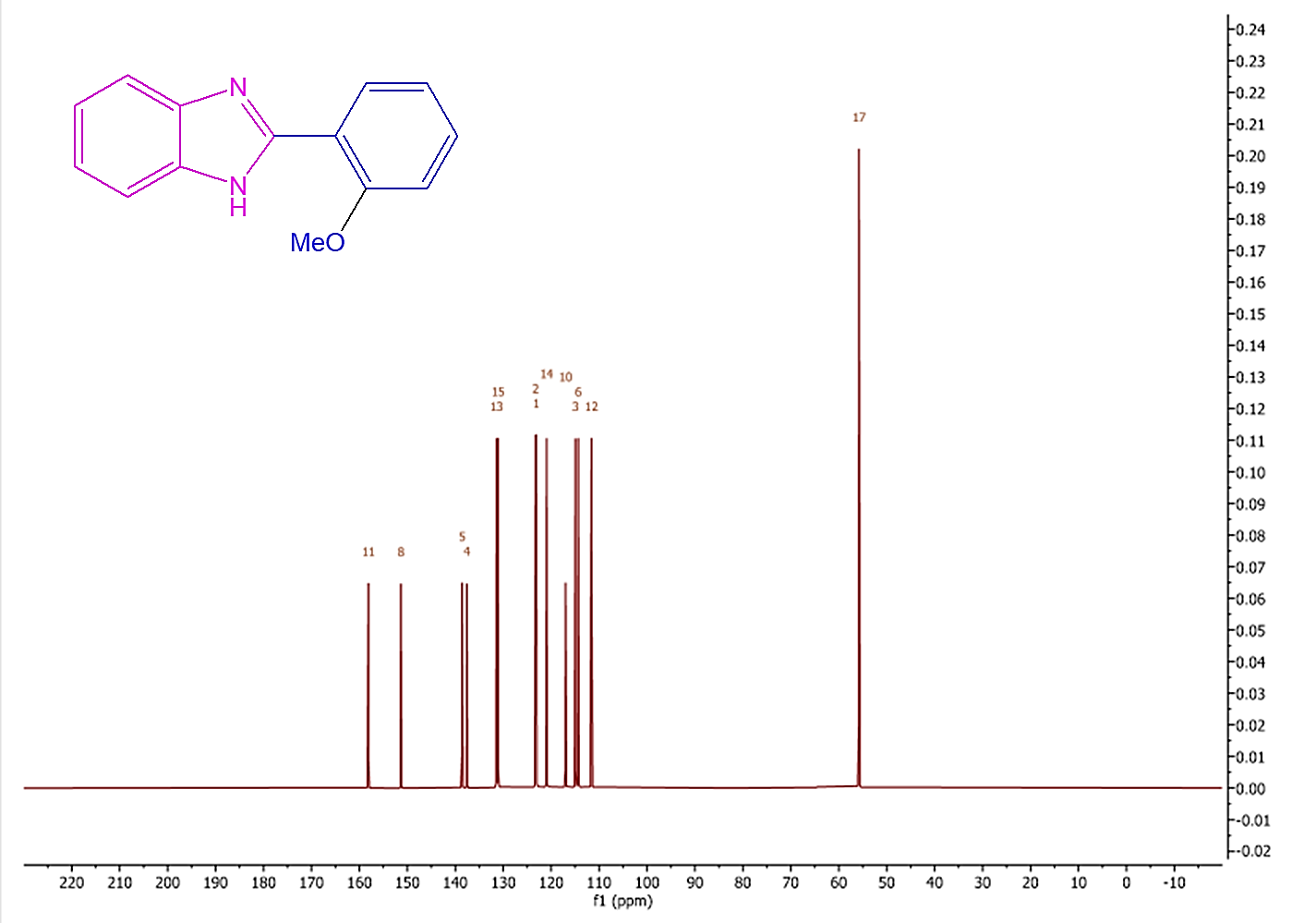


**Fig. S28:** ^13^CNMR of the 2-(2-methoxyphemyl)-1*H*-benzo[*d*]imidazole.


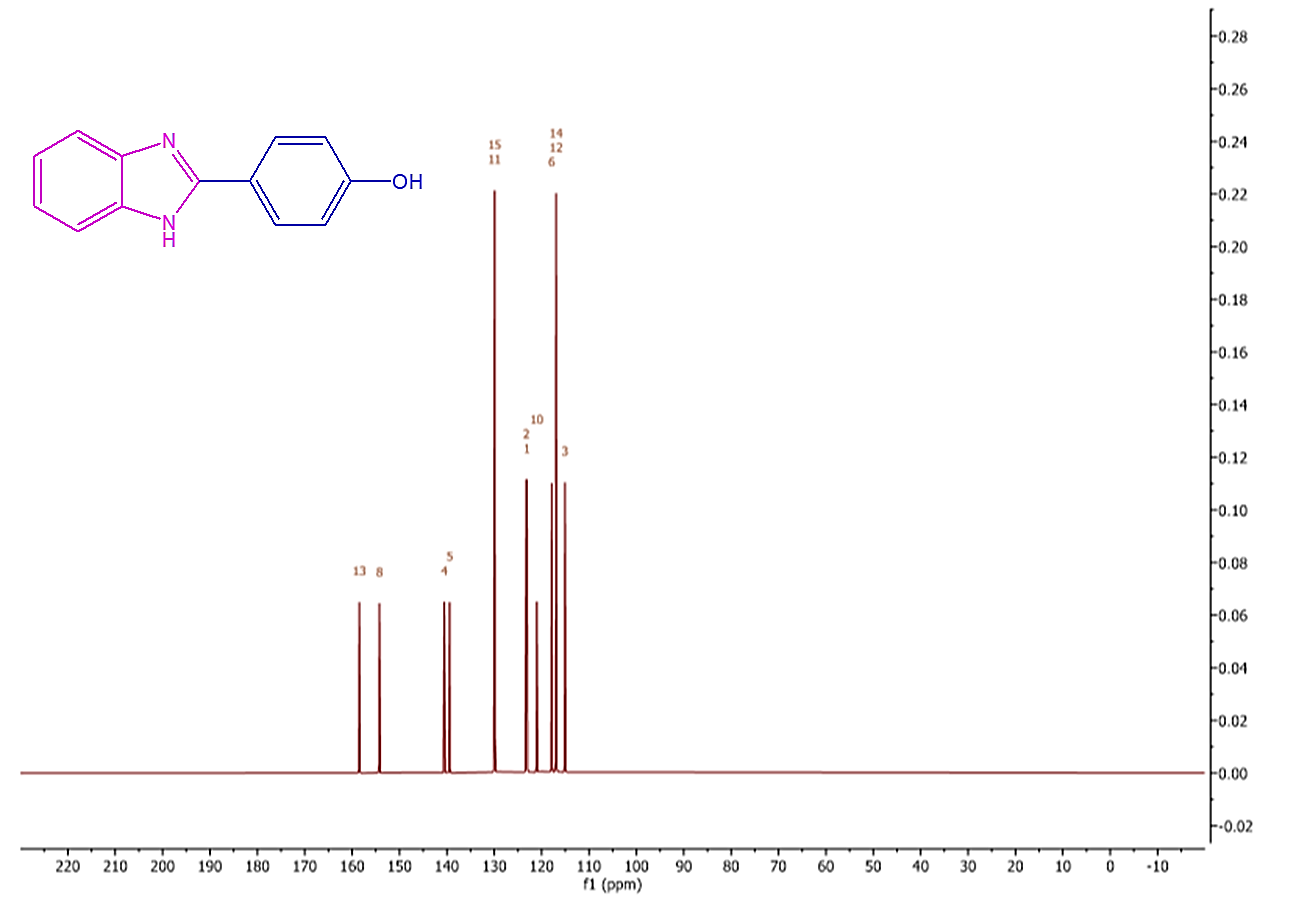


**Fig. S29:** ^13^CNMR of the 2-(1*H*-benzo[*d*]imidazole-2-yl) phenol).


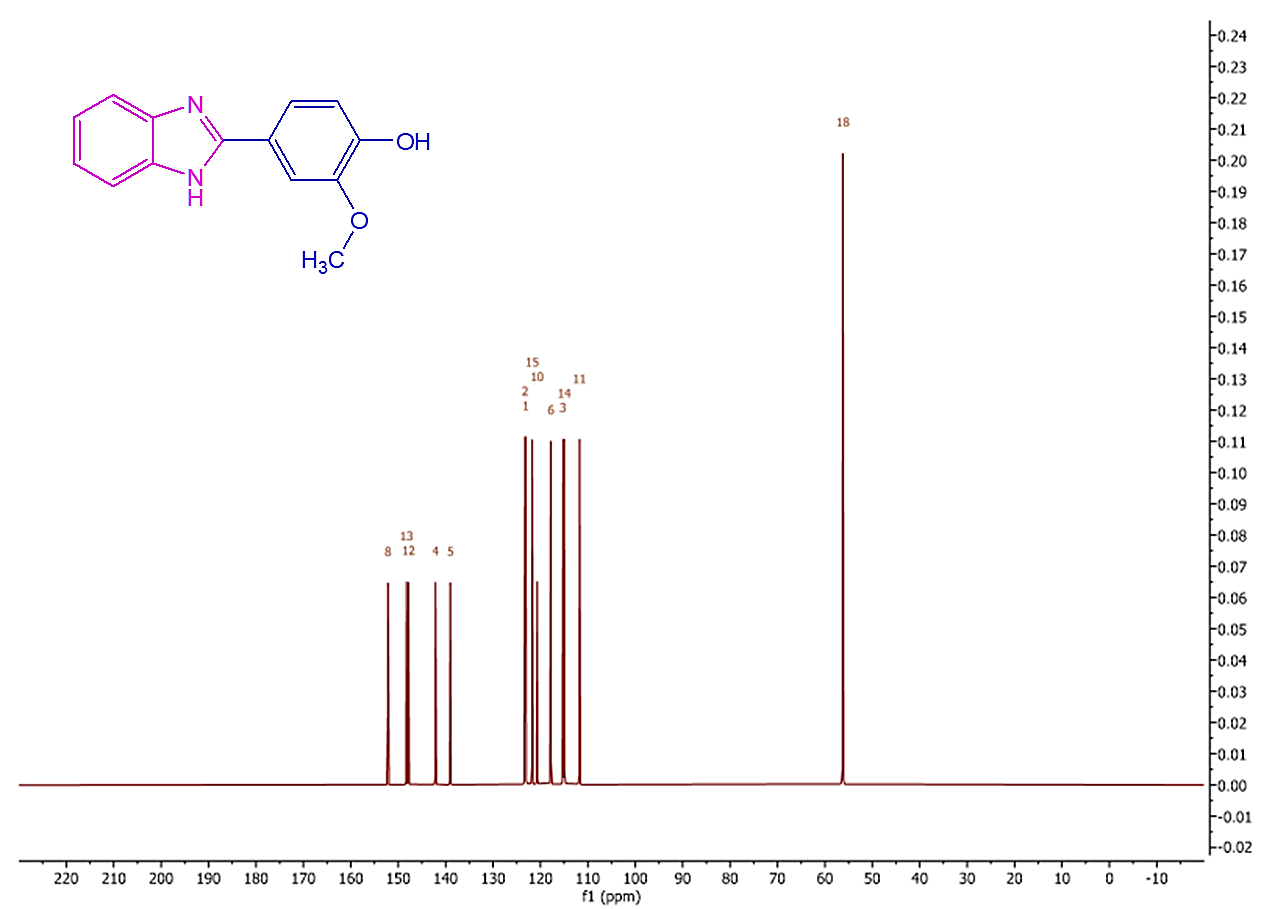


**Fig. S30:** ^13^CNMR 2-(1*H*-benzo[*d*]imidazole-2-yl) methoxyphenol).
